# The translatome of glioblastoma

**DOI:** 10.1002/1878-0261.13743

**Published:** 2024-10-17

**Authors:** Fleur M. G. Cornelissen, Zhaoren He, Edward Ciputra, Richard R. de Haas, Ammarina Beumer‐Chuwonpad, David Noske, W. Peter Vandertop, Sander R. Piersma, Connie R. Jiménez, Cornelis Murre, Bart A. Westerman

**Affiliations:** ^1^ Department of Molecular Biology University of California, San Diego La Jolla CA USA; ^2^ Department of Neurosurgery Amsterdam UMC, Location VUMC, Cancer Center Amsterdam The Netherlands; ^3^ OncoProteomics Laboratory, Cancer Center Amsterdam Amsterdam UMC The Netherlands

**Keywords:** glioblastoma, non‐coding RNA, radioresistance, radiotherapy, translatome

## Abstract

Glioblastoma (GB), the most common and aggressive brain tumor, demonstrates intrinsic resistance to current therapies, resulting in poor clinical outcomes. Cancer progression can be partially attributed to the deregulation of protein translation mechanisms that drive cancer cell growth. In this study, we present the translatome landscape of GB as a valuable data resource. Eight patient‐derived GB sphere cultures (GSCs) were analyzed using ribosome profiling and messenger RNA (mRNA) sequencing. We investigated inter‐cell‐line differences through differential expression analysis at both the translatome and transcriptome levels. Translational changes post‐radiotherapy were assessed at 30 and 60 min. The translation of non‐coding RNAs (ncRNAs) was validated using in‐house and public mass spectrometry (MS) data, whereas RNA expression was confirmed by quantitative PCR (qPCR). Our findings demonstrate that ribosome sequencing provides more detailed information than MS or transcriptional analyses. Transcriptional similarities among GSCs correlate with translational similarities, aligning with previously defined subtypes such as proneural and mesenchymal. Additionally, we identified a broad spectrum of open reading frame types in both coding and non‐coding mRNA regions, including long non‐coding RNAs (lncRNAs) and pseudogenes undergoing active translation. Translation of ncRNAs into peptides was independently confirmed by in‐house data and external MS data. We also observed that translational regulation of histones (downregulated) and splicing factors (upregulated) occurs in response to radiotherapy. These data offer new insights into genome‐wide protein synthesis, identifying translationally regulated genes and alternative translation initiation sites in GB under normal and radiotherapeutic conditions, providing a rich resource for GB research. Further functional validation of differentially expressed genes after radiotherapy is needed. Understanding translational control in GB can reveal mechanistic insights and identify currently unknown biomarkers, ultimately enhancing the diagnosis and treatment of this aggressive brain cancer.

## Graphical abstract

We analyzed the translatome landscape in glioblastoma models, revealing that ribosome profiling detected significantly more translated genes than mass spectrometry, leading to detailed insights into protein translation mechanisms. We discovered novel peptides, translated lncRNAs and pseudogenes, along with radiation effects on histones and splicing factors, enriching glioblastoma research and opening avenues for future research that may improve cancer treatment outcomes.

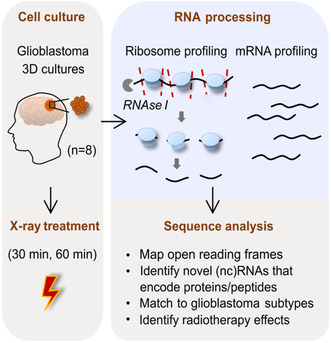

**Abbreviations**
CDS: coding sequence
circRNA: circular RNA
CL: classical
DE: differential expression
DSB: double‐strand break
ECM: extracellular matrix
GB: glioblastoma
GO: gene‐ontology
GSC: glioblastoma sphere culture
Gy: Gray
HDAC: histone deacetylase inhibitor
LC–MS: liquid chromatography–mass spectrometry
lncRNA: long non‐coding RNAs
MES: mesenchymal
miRNA: microRNA
mRNA: messenger RNA
MS: mass spectrometry
MSA: multiple sequence alignment
ncRNA: non‐coding RNA
ORF: open‐reading frame
PME: percentage of maximum entropy
PN: proneural
qPCR: quantitative polymerase chain reaction
ribo‐seq: ribosome sequencing
RNA‐seq: mRNA sequencing
RPF: ribosomal footprints
sc‐RNA‐seq: single‐cell RNA‐sequencing
shORF: short open reading frame
TCGA: the Cancer Genome Atlas Program
TMZ: temozolomide
TPM: transcript per million
uORF: upstream open reading frame

## 1 Introduction

Glioblastoma (GB) is the most common primary brain malignancy in adults and one of the most aggressive cancers [1]. Despite the standard treatment consisting of maximal safe surgical resection, radiotherapy and temozolomide (TMZ) chemotherapy, the median survival is 14.6 months only with a 2‐year survival rate of 26.5% [2]. Significant obstacles hindering the development of effective treatment include the inability of extensive tumor debulking, tumor heterogeneity [3, 4] and the existence of residual therapy resistant glioma cells that are not well defined and give rise to glioblastoma recurrence [5, 6, 7, 8].

Radiotherapy is used in the treatment of GB to improve both local control and survival. However, radiotherapy is an effective treatment method for a limited bulk of GB tumor cells, since imaging and pathological analysis have revealed that GBs typically recur within the initial radiation target volume [5]. This indicates that the remaining GB cells *in situ* are highly radioresistant. Studies have shown that glioma stem cells are resistant to radiotherapy [6] and TMZ chemotherapy [9], suggesting an important role of glioma stem cells in the development and recurrence of GB. They have the capacity for tumor generation causing the tumor to relapse [10, 11]. In addition, GSCs have migrative properties that allow them to migrate, regenerate, and invade the brain better than the differentiated cells, making it impossible to perform a complete GB resection in patients [10, 12]. The mechanisms responsible for tumor radioresistance have remained elusive and clinically effective radiosensitizers that overcome radioresistance have yet to be identified. Radioresistance is the result of activation of the DNA damage checkpoint response and increased levels of DNA repair [6]. Also, XPO1, a nuclear receptor that exports RNA to the cytoplasm, functions as a hub protein in GSCs after irradiation *in vitro* [13]. The addition of Selinexor (XPO1 inhibitor) inhibited the repair of radiation‐induced DNA double‐strand breaks, resulting in radiosensitivity enhancement.

Transcriptome sequencing has revealed the existence of thousands of unique non‐coding RNA (ncRNA) sequences within cells [14, 15, 16, 17, 18], such as microRNAs (miRNAs), circular RNAs (circRNAs), and long ncRNAs (lncRNAs) [19, 20]. ncRNAs constitute nearly 60% of the transcriptional output in human cells [21] and are considered non‐functional. Surprisingly, they have been shown to regulate cellular processes and molecular pathways in developmental and pathological contexts. Several studies have found that a large fraction of lncRNAs are associated with ribosomes and the pattern of ribosome protection indicates that lncRNAs are capable of translating short peptides (< 100 amino acids) [22, 23, 24, 25]. In addition, several lncRNAs have regulatory functions [26, 27, 28, 29], such as thymocyte differentiation factor transcript *thymoD* regulating chromatin folding and compartmentalization in T‐cells [29], X‐inactive‐specific transcript *Xist* regulating X chromosome inactivation [28] and *H19* inducing changes in IGF2 methylation [30]. However, a function for the vast majority of ncRNAs remains to be revealed.

Translational control is a key component of gene regulation. However, our understanding of its role in mammalian cells is limited. To gain a better insight, ribosome profiling [31], a method to sequence messenger RNAs (mRNAs) captured by ribosomes at a given time point in a cell, provides a comprehensive and real‐time snapshot of genes translated in a cell. Specifically, it allows the identification of multiple translated open‐reading frames (ORFs) in both coding and non‐coding genes and the discovery of novel translated genes [22, 32, 33]. Direct analysis of translation, instead of the correlation between mRNA and protein levels which is frequently poor [34], provides a more precise and complete measure of gene expression, allowing a better insight in cellular processes.

Our understanding of the cellular processes in GB and molecular pathways causing therapeutic resistance is limited. This study aims to provide a framework for understanding the translatome in GB in its natural state and after application of radiotherapy. By using ribosome profiling of patient‐derived glioblastoma spheroid culture cells (GSCs, glioma stem‐like cells isolated from GB tissue), we elucidated the translational landscape of eight different GSC cultures recapitulating the three major GB subtypes. On a global level, we show compliance between transcription and translation by confirming that the three major GB transcriptional subtypes are selected in their respective translational profile. We show that ribosome sequencing analysis proved a deeper view of translation processes than MS analysis which could therefore provide a finer‐grained view of biological processes, also when compared to transcriptional analyses. Furthermore, we identified gene families that show immediate translational differences after irradiation, implicating translational regulation as a result of radiotherapy.

In addition, our developed bioinformatic workflow allowed us to discover not yet identified ncRNAs. We discovered that numerous ncRNAs are actually translated in GB and show that at least a subset of the newly identified genes encodes functional peptides as shown by MS analysis. Overall, we could validate that a substantial amount of riboseq‐identified peptides are present in the Human PeptideAtlas and we have performed an in‐house validation that yielded a handful of validated peptides. Together, these data provide a comprehensive overview of the translational landscape of GB, in both normal and irradiated cells and accommodate a rich resource of translation in GB for future research.

## 2 Materials and methods

### 2.1 Source of human primary glioma sphere cultures and ethical statement

All methods were carried out following relevant guidelines. Four primary glioma sphere cultures (GSCs; GSC34, GSC2, GSC20, and GSC28 respectively; Table S1) were provided by E. Sulman [35] (NYU Langone Health, New York, NY) and derived at the M.D. Anderson Cancer Center or University Medical Center Groningen. The use of human tumor tissue samples was conducted under approval by the Institutional Review Board at the University of Texas, M.D. Anderson Cancer Center or by the Medical Ethical Committee at the University Medical Center Groningen. The use of tissues for experiments involving isolation of GSCs and DNA and RNA isolation was exempt from requiring consent as per the MD Anderson Cancer Center Institutional Review Board. Primary GSCs from the University Medical Center Groningen were obtained from tumor material after routine diagnostics, coded according to the National Code for the Good Use of Patient Material, and were exempt from written informed consent. Four independently obtained primary GSCs (VU591, VU593, VU598 and VU609, respectively; Table S1) were derived by F.M.G.C. and B.A.W. at the Amsterdam University Medical Center/Cancer Center Amsterdam, the Netherlands and coded according to the National Code for the Good Use of Patient Material and were exempt from written informed consent. Tissues were collected between January 2015 and December 2018. Tumor sample collection at MD Anderson was performed under protocol #LAB03‐0687, approved by the institutional review board, after written informed consent was obtained from the patients. Tumor samples at the Amsterdam UMC and UMC Groningen were collected under the National policy on further use of bodily material CCMO #7:467. All research was performed in accordance with the declaration of Helsinki.

### 2.2 Human cell culture experiments

All primary GSC cell cultures derived from GB patient tumor tissue were performed at 37 °C under 5% CO_2_. All cell lines were tested to exclude the presence of *Mycoplasma* infection. GSCs were cultured in artificial Neurobasal‐A‐Medium (NBM; 11540366; Gibco, Paisley, Scotland, UK) supplemented with 1x B27 supplement without vitamin A (11530536; Gibco), 1× N2 supplement (12013479; Gibco), 1% Glutamax (11574466; Gibco), 20 ng/ml human EGF (AF‐100‐15; PeproTech, Thermo Fisher, Carlsbad, CA, USA), 20 ng/ml human FGF basic (100‐18B; PeproTech), 0.1% heparin (07980; StemCell, Vancouver, Canada), and 1× penicillin/streptomycin (15140122; Gibco).

#### 2.2.1 Radiotherapy for ribosome sequencing

Cells were seeded at 10 × 10^6^ in a T175 flask and cultured overnight. Cells were irradiated once at room temperature with 2 Gy using a Mark I Cesium‐137 source (JL shepherd and Associates, San Fernando, CA, USA). and lysed at respectively 0‐, 30‐, and 60‐min post‐irradiation, followed by detergent lysis with flash‐freezing in liquid nitrogen for ribosome profiling and mRNA‐seq.

#### 2.2.2 Radiotherapy dose–response

Spheroids were irradiated at room temperature using a Cobalt‐60 source at a dose rate of 516 Gy·h^−1^ (Gammacell 220; Atomic Energy of Ottawa, Canada). 3D spheroid cultures were formed by plating cells at 3000 cells/well in ultra‐low attaching repellent plates (#3471; Corning, Glendale, AZ, USA) [36]. Four days were allowed for spheroid formation. Images were automatically captured on a Leica DMI3000 microscope (Leica, Deer Park, IL, USA) using universal grab 6.3 software (DCILabs). Spheroid sizes were determined using scratch assay 6.2 (DCILabs, Nashville, TN, USA). Spheroid volumes were quantified as a function of time which allows analyses of size reduction and growth rate in time.

### 2.3 Ribosome profiling

Ribosome profiling on GSCs (*n* = 8, 3 conditions per subject) was performed using the latest version of the ribosome profiling protocol optimized for use on GSC samples [31]. In brief, the Ribo‐seq procedure on GSC samples was carried out as follows: after washing the cells with ice‐cold PBS and centrifugation cells were lysed for 10 min on ice in 1 mL lysis buffer consisting of 20 mm Tris pH 7.5, 150 mm NaCl, 5 mm MgCl2, 100 μg·mL^−1^ cycloheximide, 1% Triton X‐100, 10 U·mL^−1^ Turbo DNase I and nuclease free H_2_O. Each sample was triturated 10 times through a 26 G needle on ice to dissociate cell clumps. Next, samples were centrifuged at 20 000 ***g*** for 10 min at 4 °C to pellet cell debris. A total of ±25 μg lysate per sample was used and processed according to the protocol with a few modifications [31]. For mRNA digestion, 7 U RNase I (10 U·μL^−1^ by Epicentre definition) was used and ribosome footprints (RPFs) were purified using Microspin S‐400 Columns (GE Healthcare, Fairfield, CT, USA) according to protocol. Next, RPFs were purified using acid‐phenol chloroform (AM9720; Invitrogen, Thermo Fisher, Carlsbad, CA, USA). Purification of linker‐ligated RPFs was accomplished by running a 10% polyacrylamide TBE‐Urea gel (3450089; Bio‐Rad, Hercules, CA, USA). Reagents used for ribosome profiling are summarized in Table S1.

For all samples, ribosome profiling library size distributions were checked on a BioAnalyzer 2200 using a high‐sensitivity DNA assay (5067‐5582; Agilent, Santa Clara, CA, USA). Samples were multiplexed and sequenced (1 × 75 bp single end, minimum 50 million reads per sample) on an Illumina HiSeq 2500, as previously described [31]. Samples were always processed in batches of a maximum of 12 samples to avoid sample processing bias.

### 2.4 RNA‐seq library preparation

For RNA‐seq, total RNA (2–5 μg) was purified by Trizol (15596018; Invitrogen) extraction from the same cell lysates processed for ribosome profiling, ribosomal RNA (rRNA) depleted and converted into deep sequencing libraries. RIN scores were measured on a BioAnalyzer 2200 using the RNA 6000 Nano assay (5067‐1511; Agilent). mRNA‐seq libraries were generated from high‐quality RNA (average RNA Integrity Number (RIN) of 9.5); Table S1. Libraries were multiplexed and sequenced on an Illumina HiSeq4000 producing paired 2 × 50 bp reads, ±20 million reads per sample (Table S1).

### 2.5 Ribosome profiling and RNA‐seq analysis

#### 2.5.1 Ribosome profiling

Reads are processed and mapped in the following steps:

1. Adaptor sequences are removed with cutadapt 1.14 with the following settings: ‐u 1 ‐m 15 ‐e 0.2 ‐O 7 ‐‐discard‐untrimmed.
2. Reads are separated based on library barcode by cutadapt 1.14 with option ‐e 0.2.
3. Unique Molecular Identifiers (UMIs) are extracted and clustered using umi_tools 0.5.5.
4. Reads are mapped to hg38 by bowtie 1.2.2 with the following setting: ‐‐chunkmbs 2000 ‐k 1 ‐‐best ‐S.
5. UMIs are collapsed to remove sequencing errors and PCR errors. Umitools dedup was used for this purpose.
6. rRNA reads are removed based on coordinates of rRNA in GENCODE reference human genome (hg) 38 version 30.

RibORF v1.0 is used to determine the quality of the samples [37]. For each sample, we then select reads by read length to best represent the 3‐nt periodicity of canonical ORFs. RibORF is then used to predict ORFs by using the known ORFs as training sets. We use the RibORF‐output *P*‐value cutoff of 0.7 (false‐positive rate 0.0095, true‐positive rate 0.936) to select potential novo‐ORFs.

#### 2.5.2 Preparation of RNA‐seq human genome annotation reference file including novel ncRNAs

To capture the complete GSC transcriptome including not yet annotated novel ncRNAs we created *de novo* transcriptome assemblies used as reference annotations for mapping RNA‐seq and ribosome profiling data. First, all RNA‐seq fastq files were mapped to the hg38v30 reference annotation file using star (STAR_2.5.1b, with option –outSAMunmapped Within –outFilterMultimapNmax 10 –outFilterMultimapScoreRange 1) and possible transcripts were identified with stringtie (1.3.3b). Next, all created GTF files were merged with cuffmerge v1.0.0. cuffcompare v2.2.1 was used to compare the generated GTF file with the reference annotation file and label the known transcripts. Finally, we labeled the unannotated transcripts as novo‐ncRNAs.

#### 2.5.3 Quantification of Ribo‐seq and mRNA‐seq data

mRNA‐seq reads were quantified by featurecount v1.5.1 with the newly assembled transcriptome. Ribo‐profiling reads were quantified by our laboratory‐developed code (https://github.com/Arthurhe/RiboReadCountByFrame). The code counts only transcripts which contain both a start codon and a stop codon. The transcripts should encode a minimum of at least 3–8 amino acids. One gene can have multiple transcripts or transcript variants. The code finds only the transcripts of a gene with both start and stop codons in an ORF. In this way, it can find all possible peptide‐encoding transcripts of a gene in a sample. In this way, we can quantify the number of reads per ORF and gene.

### 2.6 Data analysis

#### 2.6.1 Read densities around start and stop codons of canonical ORFs

RibORF groups read based on fragment length, and checks the distance between 5′ ends of the reads around the start and stop codons of canonical ORFs of mRNAs. The length of ribosome‐protected fragments can range from 17 to 34 nucleotides (nt). Fragment lengths showing clear 3‐nt periodicity with a high percentage of reads in first nucleotides of codons (> 50%) from start to stop codons of canonical ORFs per sample were selected for further analysis.

#### 2.6.2 Comparison of ribo‐ and mRNA‐seq data with proteome data

To compare genes identified with ribosome profiling (translatome) and RNA sequencing (transcriptome) with protein data (proteome), we used published mass‐spec data [38] of respectively GSC2, GSC20, GSC28, and GSC34. In this paper, foldchanges relative to M37 (a mixed control sample containing equal protein from each of the GSC cell lines studied) were used to compare and characterize GSCs. Genes with a foldchange of > 0 in one or more GSC samples were selected for comparison with our developed ribosome sequencing and RNA sequencing data of our identical GSC samples under normal conditions (*t* = 0). From our datasets, we selected genes in respectively mRNAseq (transcripts per million (tpm) > 1) and Ribo‐seq (tpm > 1) to compare the gene lists of transcriptome, translatome, and proteome data, respectively. tSNE dimension reduction analysis of transcriptome and translatome data was performed using the R2 platform (r2.amc.nl).

#### 2.6.3 ORF types and start codons identified in GSCs

RibORF generates a summary of ORF types and corresponding start codons (ATG, CTG, GTG, TTG, ACG) identified in GSCs. ORF types were categorized into three main groups, respectively, main‐ORF, alt‐ORF and candidate‐ORF [39]. The correlation of ORF types between cell lines was calculated as the number of specific ORF types divided by the total number of ORFs per cell line. Next, hierarchical clustering of cell lines and ORF types was performed.

#### 2.6.4 Coding potential of candidate‐ORFs

Fractions of reads in respectively first, second, and third nucleotide in codons were extracted from RibORF, as well as PME value, predicted *P*‐value and ORF lengths of canonical, non‐coding and uORF ORF types.

#### 2.6.5 Non‐coding genes transcribed and translated in GSCs

To obtain an overview of transcribed and translated non‐coding genes present in GSCs, we selected genes in respectively mRNAseq (tpm > 1) and Ribo‐seq (tpm > 1) and matched biotype information of genes. The following gene types were classified as non‐coding genes: 3′ overlapping ncRNA, antisense, lincRNA, bidirectional promoter lncRNA, macro lncRNA, non‐coding, processed transcript, sense intronic, sense overlapping, miscRNA, miRNA, snoRNA, Mt rRNA, Mt tRNA, scRNA, snRNA, scaRNA, pseudogenes (transcribed (un)processed, polymorphic, (transcribed) unitary, and unprocessed pseudogenes, respectively) and *de novo* ncRNA. Next, gene types were subcategorized in four groups, respectively, new non‐coding RNAs (*de novo* ncRNA), lncRNAs (antisense, bidirectional promoter lncRNA, lincRNA, non‐coding, processed transcript, sense intronic, and sense overlapping), short ncRNA (miRNA, snRNA, snoRNA, scaRNA, mt tRNA, miscRNA), and pseudogenes (transcribed (un)processed, polymorphic, (transcribed) unitary, and unprocessed pseudogenes).

#### 2.6.6 Multiple sequence alignment non‐coding ORFs

From the non‐coding gene list, all possible ORFs per gene were selected and duplicates were removed. Corresponding DNA sequences from the ORFs were obtained from Ensembl. ORF peptide sequences with stop codons were deleted from the analysis (a total of 369 ORFs). Multiple sequence alignment of non‐coding ORFs (a total of 1661 ORFs) was performed by clustal omega (EMBL‐EBI, https://www.ebi.ac.uk/jdispatcher/msa/clustalo).

#### 2.6.7 Mass spectrometry analysis summary

GB cell lines GSC2, GSC34, VU593, and VU598 were cultured to 80% confluency. These cell lines were enriched for protein products of (novo) non‐coding RNA using C8 hydrophobic interaction chromatography. To restrict the protein molecular weight (Mw) to a maximally 30 kDa, the C8 fractions were applied to gel electrophoresis. The proteins with an Mw < 30 kDa were cut out of the gel‐lane and digested in‐gel. Subsequently, to deepen the proteomic analysis, each > 30 kDa sample was fractionated into five fractions using high‐pH reversed‐phase chromatography. LC–MS/MS data were searched against a combination of the SwissProt human FASTA file and two Ribo‐Seq derived custom FASTA files.

#### 2.6.8 Mass spectrometry sample preparation

After washing with PBS, 10 mg was lysed in lysis buffer (9 m urea, 20 mm HEPES pH 8.0, 1 mm sodium orthovanadate, 2.5 mm sodium pyrophosphate, 1 mm beta‐glycerophosphate), followed by three cycles of sonication (MSE soniprep 150). Afterward, samples were cleared by centrifugation (4500 ***g***, 20 min, room temperature). Cleared lysate was acidified to pH 3.5 using formic acid and enriched for small proteins and desalted using a C8 cartridge (Sep‐Pak 500 mg, WAT054525; Waters, Milford, MA, USA). Proteins were eluted in 80% ACN/1% HAc. After drying in a speed‐vac, 35 μg desalted lysate was dissolved in NuPAGE sample buffer and applied to a NuPAGE MES gradient gel. The lower half of the gel containing proteins in the Mw range 4–30 kDa was excised and cut into 1 mm^3^ cubes, reduced in 10 mm DTT (dithiothreitol) and alkylated in 54 mm IAM (iodoacetamide). After washing in 50 mm ABC (ammonium bicarbonate buffer pH 7.8) and ABC/ACN (1 : 1 v/v), gel cubes were dried and incubated overnight at 25 °C with 6.3 ng·mL^−1^ sequencing‐grade trypsin (V5111; Promega, Madison, WI, USA). Peptides were extracted using 1% formic acid (1×) and 50% ACN/5% formic acid (2×), pooled per sample and stored at −20 °C. Peptides were fractionated into five fractions using high‐pH reversed‐phase in STAGE‐tips (five layers 3M EMPORE SDB‐XC, 1 mm discs in a 200 μL tip). Peptides were dried in a speed‐vac and dissolved in 50 μL 1% NH_4_OH. STAGE‐tips were activated with 50 μL ACN, washed in 0.1% TFA and equilibrated in 1% NH_4_OH, followed by application of the sample. In all, 15 fractions were collected at 2.5% ACN (in 1% NH_4_OH) increments. These 15 fractions were concatenated into five fractions with similar hydrophobicity distributions.

#### 2.6.9 LC–MS/MS peptide separation

The methods used for peptide separation have also been published previously [40]. Peptides were separated using an Ultimate 3000 nanoLC‐MS/MS system (Thermo Fisher Scientific, Carlsbad, CA, USA) equipped with a 50 cm × 75 μm ID Acclaim Pepmap (C18, 1.9 μm) column. After injection, peptides were trapped at 3 μL·min^−1^ on a 10 mm × 75 μm ID Acclaim Pepmap trap at 2% buffer B (buffer A: 0.1% formic acid (Fisher Scientific), buffer B: 80% ACN, 0.1% formic acid) and separated at 300 nL·min^−1^ in a 10–40% buffer B gradient in 110 min (140 min inject‐to‐inject) at 35 °C. Eluting peptides were ionized at a potential of +2 kVa into a Q Exactive HF mass spectrometer (Thermo Fisher Scientific). Intact masses were measured from *m/z* 350–1400 at resolution 120 000 (at *m/z* 200) in the Orbitrap using an AGC target value of 3E6 charges and a maxIT of 100 ms. The top 15 for peptide signals (charge‐states 2+ and higher) were submitted to MS/MS in the HCD (higher‐energy collision) cell (1.4 amu isolation width, 26% normalized collision energy). MS/MS spectra were acquired at resolution 15 000 (at *m/z* 200) in the orbitrap using an AGC target value of 1E6 charges, a max of 64 ms, and an underfill ratio of 0.1%, resulting in an intensity threshold for MS/MS of 1.3E5. Dynamic exclusion was applied with a repeat count of 1 and an exclusion time of 30 s.

#### 2.6.10 Mass spectrometry data processing protocol

Protein identification was performed using maxquant 2.0.3.0 software using the Swissprot *Homo sapiens* proteome FASTA file (downloaded January 2021, canonical and isoforms, 42 383 entries) and a Ribo‐seq derived dedicated non‐coding and novo non‐coding FASTA file. Cysteine carbamidomethylation (Cys, +57.021464 Da) was treated as fixed modification and methionine oxidation (Met, +15.994915 Da) and N‐terminal acetylation (N‐terminal, +42.010565 Da) as variable modifications. Searches were performed using semi‐tryptic specificity to accommodate peptides with a (single) non‐tryptic end at the N‐ or C‐terminus, encountered with high frequency in the novo non‐coding and non‐coding FASTA files. Peptide precursor ions were searched with a maximum mass deviation of 4.5 p.p.m. and fragment ions with a maximum mass deviation of 20 p.p.m. Peptide and protein identifications were filtered at an FDR of 1% using the decoy database strategy. The minimal peptide length was 7 amino‐acids and the minimum Andromeda score for modified peptides was 40 and the corresponding minimum delta score was 6. Searches were performed against the three FASTA files combined and against the non‐coding and novo FASTA files alone. Identification of non‐coding and novo peptides was assessed manually using the maxquant viewer.

#### 2.6.11 Mass spectrometry validation using external data

In addition to the mass spec analysis as outlined above, we validated the translation of non‐coding RNAs using external data, available through the Human PeptideAtlas [41] which contains 3 945 444 distinct peptides (version 01‐2024). Analysis was done using the online portal at https://db.systemsbiology.net/sbeams/cgi/PeptideAtlas/SearchProteins. This validation resulted in matching peptides (substitutions are shown in the lower case, see Table S2); predicted peptides (indicated by “CONTRIB”) or failed to be detected (UNMAPPED).

#### 2.6.12 Non‐coding RNA expression confirmation

To confirm that non‐coding RNAs as detected by ribosome profiling are transcribed, we isolated total RNA using TRIzol™ (Cat #15596018; Thermo Fisher Scientific) from respectively GSC34 and VU598 cell lines. First, strand cDNA was generated using SuperScript™ III Reverse Transcriptase (Cat #18080044; Thermo Fisher) according to the manufacturer's instructions. To determine whether RNA was present in samples of the VU598 and GSC34 cell lines, reactions were performed in the presence and absence of reverse transcriptase, where in the latter case the signal in the qPCR will be caused by primer‐dimers or amplification of DNA from contaminating genomic DNA. We performed qPCR in technical triplicates where each reaction matched 27 ng of isolated RNA, which was subsequently amplified with HOT FIREPol® EvaGreen^®^HRM Mix (ROX) (Cat #08‐33‐00020; Solis Biodyne, Tartu, Estonia) according to the manufacturer's instructions. Primers are shown in Table S1. After the PCR, the cycle threshold (*C*_t_) difference between reactions in the presence and absence of reverse transcriptase was determined and plotted as histograms showing the magnitude and indicating the statistical significance.

#### 2.6.13 Single‐cell analysis of ncRNAs

Single‐cell sequencing data of Neftel et al. [42] were analyzed using the online bioinformatic platform r2 (r2.amc.nl). First, the single‐cell data (*n* = 7930 cells) were processed using tSNE dimension reduction using standard settings. Cellular populations with similar RNA expression were detected using DBSCAN using the following settings: Epsilon: 5.95 and Min pts: 80. Normal cells could easily be identified because these formed cellular clusters shared between patients. This was seen for T‐cells (identified by CD8A expression), macrophages and microglia (identified by CD68/CD168 expression) as well as oligodendrocytes (identified by OLIG2 expression). The remaining cells showed individual clusters representative of single patients and were considered pure tumor cells. Non‐coding RNAs identified by ribosome‐profiling were analyzed for their expression in the single‐cell clusters of normal cells as well as tumor cells.

#### 2.6.14 Quantification and statistical analysis

Statistical analyses were performed by r (version 4.0.3, https://www.r‐project.org/). Statistical parameters such as the value of *n* and significance level (*P < 0.05, **P < 0.01, ***P < 0.001 and ****P < 0.0001) are reported in the figures and/or the figure legends. The “*n*” represents culture sample numbers, the number of genes or ORF numbers. Statistical parameters used to indicate DE were derived from deseq2, or otherwise the type of statistical test (e.g., *t*‐test) is annotated in the figure legend. For the qPCR confirmation of non‐coding RNAs, *P*‐values were calculated using a *t*‐test with Bonferroni multiple testing correction for 34 genes. In case no *C*_t_ value was obtained before cycle 40, a value of 40 was chosen for a one‐sample *t*‐test.

#### 2.6.15 Gene ontology analysis and MSigDB gene signatures

Gene ontology analysis on groups of genes identified from Ribo‐seq analysis (excluding the newly identified genes) was completed using the Functional Annotation Tool in the Database for Annotation, Visualization and Discovery (DAVID) [43, 44]. The data collected from the gene‐annotation enrichment analysis search included gene groups identified in a biological process, cellular compartment or molecular function. Functional annotation terms with *P*‐value < 0.0001 were included in the article. To generate gene expression signatures to distinguish proneural, mesenchymal, and classical GB subtypes, RNAseq data were converted to *z*‐values. Gene set enrichment signatures were obtained from The Molecular Signatures Database (MSigDB, www.gsea‐msigdb.org/) and used to calculate the average *z*‐value for each of the eight cell lines. The average *z*‐values per gene set were plotted per cell line for all three gene signatures, showing that they are not necessarily mutually exclusive. *K*‐means clustering and heatmap visualization were generated using the r2 platform. Default settings were used with the Color scheme BuYlRd [7], and the distance measure Euclidean distance was used. Other heatmaps were generated using complexheatmap version 2.7.3; https://github.com/jokergoo/ComplexHeatmap, gplots version 3.1.1; https://www.rdocumentation.org/packages/gplots/versions/3.1.3 or Microsoft Excel.

#### 2.6.16 DepMap phenotypic analysis of non‐coding RNAs

Data from the RNAi Achilles+DRIVE+Marcotte, DEMETER2 study [45], present in the DepMap database (https://depmap.org/portal/), were analyzed for lethal phenotypes of the identified non‐coding RNAs (*n* = 58 genes analyzed in *n* = 65 cell lines). Lethal phenotypes are assessed through a gene dependency score, where a negative value is reflective of viability loss after the knockdown of the respective target gene. Effects were assessed as a score as well as an interpretation in relation to other genes using the classifications “Weak lethal effect,” “No effect,” and “Weak growth advantage.” RNA expression of the respective analyzed genes was based on Bhat et al. (GSC models) [46], Neftel et al. [42] (single cells, excluding non‐cancer cells), TCGA‐GBM data [47] (bulk tumors), and Sanger GDSC data [48] (classical GBM cell lines), using the online bioinformatic platform r2.

## 3 Results

### 3.1 Detection of active translation in glioblastoma sphere cultures using Ribo‐seq

**Fig. 1:**
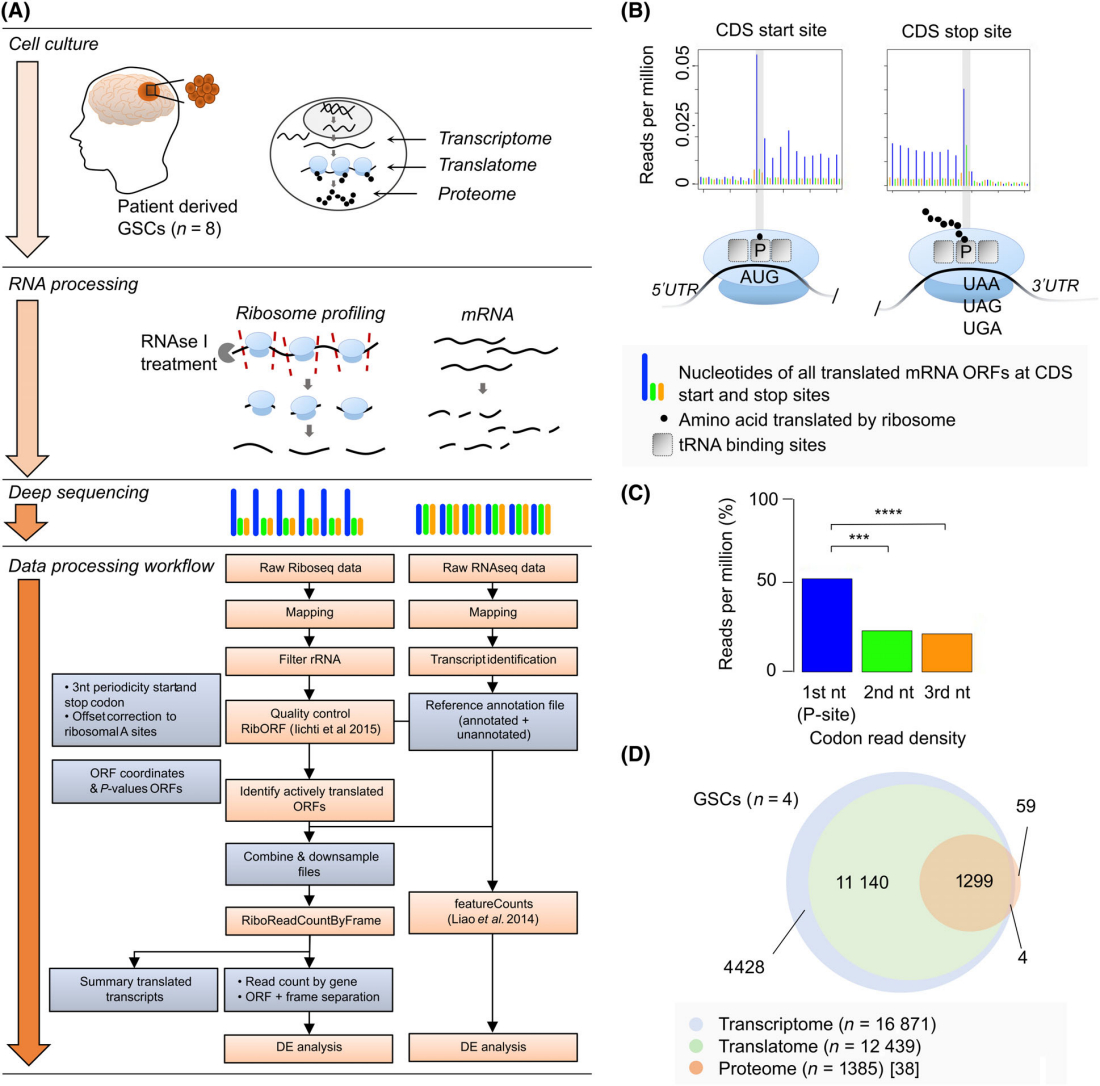
Outlining procedure for ribosome profiling in eight glioblastoma patient‐derived GSCs. (A) Schematic overview experimental approach. GSCs were analyzed with ribosome profiling and mRNA sequencing. With ribosome profiling we were able to capture actively translated genes at a given time point and mRNA sequencing was used to map the identified open reading frames (ORFs). The lower part shows the flowchart of computational analysis of Ribo‐seq data. RibORF [37] was used to filter mRNA sequences being translated by ribosomes. RiboReadCountByFrame is our laboratory‐developed bioinformatic script to identify newly translated ncRNAs and to generate gene count numbers per translated gene and consists of the following steps; first, unnecessary adaptor sequences are removed, followed by sorting based on specific barcode sequences for tracking experimental branches. Unique Molecular Identifiers are then extracted to cluster similar genetic sequences, which are mapped to a standard human genome to identify their location. Redundant sequences are filtered out, and ribosomal RNA is removed. After ensuring sample quality, ORFs are identified, and potential new protein‐building regions are predicted. In preparing the mRNA‐seq reference file, genetic mapping to a standard human genome is conducted to create a comprehensive list of genetic instructions. This includes identifying transcripts, merging data with known genetic sequences, and discovering new types of genetic sequences [129]. Quantifying genetic data involves counting the number of reads associated with each gene using the newly assembled transcriptome for mRNA‐seq and specialized code for ribosome profiling. Data analysis includes examining patterns of genetic reads around start and stop codons, comparing ribosome profiling and RNA sequencing data with protein data, identifying different types of protein‐building regions and start codons, assessing coding potential of newly identified regions, and exploring non‐coding genes that are transcribed. DE, differential expression; GSC, glioma sphere cells; mRNA, messenger RNA; *n*, represents the number of patient derived GSC samples; nt, nucleotide; ORF, open reading frame; RNAseq, RNA sequencing; rRNA, ribosomal RNA. The mRNA and ribosome profiling data are based on one technical replicate per GSC sample. (B) Read densities around start and stop codons of canonical ORFs. Good quality ribosome footprints show a clear three‐nucleotide periodicity after nuclease digestion and follow a typical high‐low‐low number of reads pattern (depicted in blue‐orange‐green), arising from the translocation of ribosomes along the mRNA one codon at a time. CDS, coding sequence; ORF, open reading frame; P, P‐site (i.e., binds to the tRNA holding the growing polypeptide chain of amino acids); t‐RNA, transfer‐RNA; UTR, untranslated region. (C) Percentage of reads in each nucleotide (nt) of codons, respectively first (P‐site), second, and third nucleotide of all ribosome profiling samples combined, illustrating the clear three‐nucleotide periodicity expected from ribosome footprints after appropriate nuclease digestion. *P*‐values were determined by Student's *t*‐test (***P value < 0.001; ****P value < 0.0001). (D) Identified transcriptome (mRNA sequencing) and translatome (ribosome profiling) genes in four GSC cultures under normal circumstances (*n* = 4, i.e., GSC34, GSC2, GSC20, and GSC28), compared with previously published proteome (mass spectrometry) data of these four identical cell lines [38]. The majority of identified genes show overlap in all three levels of gene expression. Ribosome profiling revealed 10 times as many translated genes compared to mass spectrometry (12 439 genes vs. 1385 genes). Proteome, translatome, and transcriptome data are based on one technical replicate per GSC.

To study mRNA expression and translation in GSCs, we applied mRNA sequencing (mRNA‐seq) and ribosome sequencing (Ribo‐seq) to 8 GB patient‐derived GSCs (Fig. 1A, Fig. S1A,B, Table S1). Sequenced ribosomal footprints (RPFs) map to canonical open reading frames (ORFs) from coding sequence (CDS) start to CDS stop site and display the 3‐nucleotide (3‐nt) movement characteristic of actively translating ribosomes (Fig. 1B,C). To obtain translated sequences in GSCs, we created a *de novo* transcriptome assembly and performed a search for actively translated ORFs using RibORF [37]. Next, ribosome profiling reads were quantified for differential expression analysis.

We compared our transcriptome and translatome data of four GSCs, respectively GSC2, GSC20, GSC28, and GSC34, with the most recent proteome data to date on identical cell lines [38]. Our transcriptome analysis found a total of 16 871 individual genes being translated in these cell lines (Fig. 1D). Ribosome sequencing revealed a total of 12 439 genes, that is, 74% of the transcriptome were undergoing protein synthesis. In comparison, translation detection by MS detected around 8% (1385 individual genes) genes translated. Ribosome sequencing found 89% more genes compared with MS, showing a much better sensitivity for RNA, probably due to its lower complexity compared to the fully translated, folded, and compartmentalized proteins as being detected by MS. Additionally, not all protein sizes can be detected using MS, the overlap of detected genes is directly dependent on the depth of the MS analysis performed and highly expressed proteins disturb the detection of lower‐expressed proteins by MS. Furthermore, ribosome sequencing has a broader overlap with MS compared to mRNA sequencing. Of the total transcriptome, 26% (4428 genes) were not actively translated under normal cell circumstances. To conclude, translatome analysis by ribosome sequencing gives better insights into active intracellular processes compared with transcriptome analysis. In addition, ribosome sequencing gives a broader insight of genes being translated compared with MS, because of notable differences in methodology and analytical technique.

### 3.2 Ribosome profiling reveals a new perspective on translated open reading frames in both coding and “non‐coding” regions

**Fig. 2:**
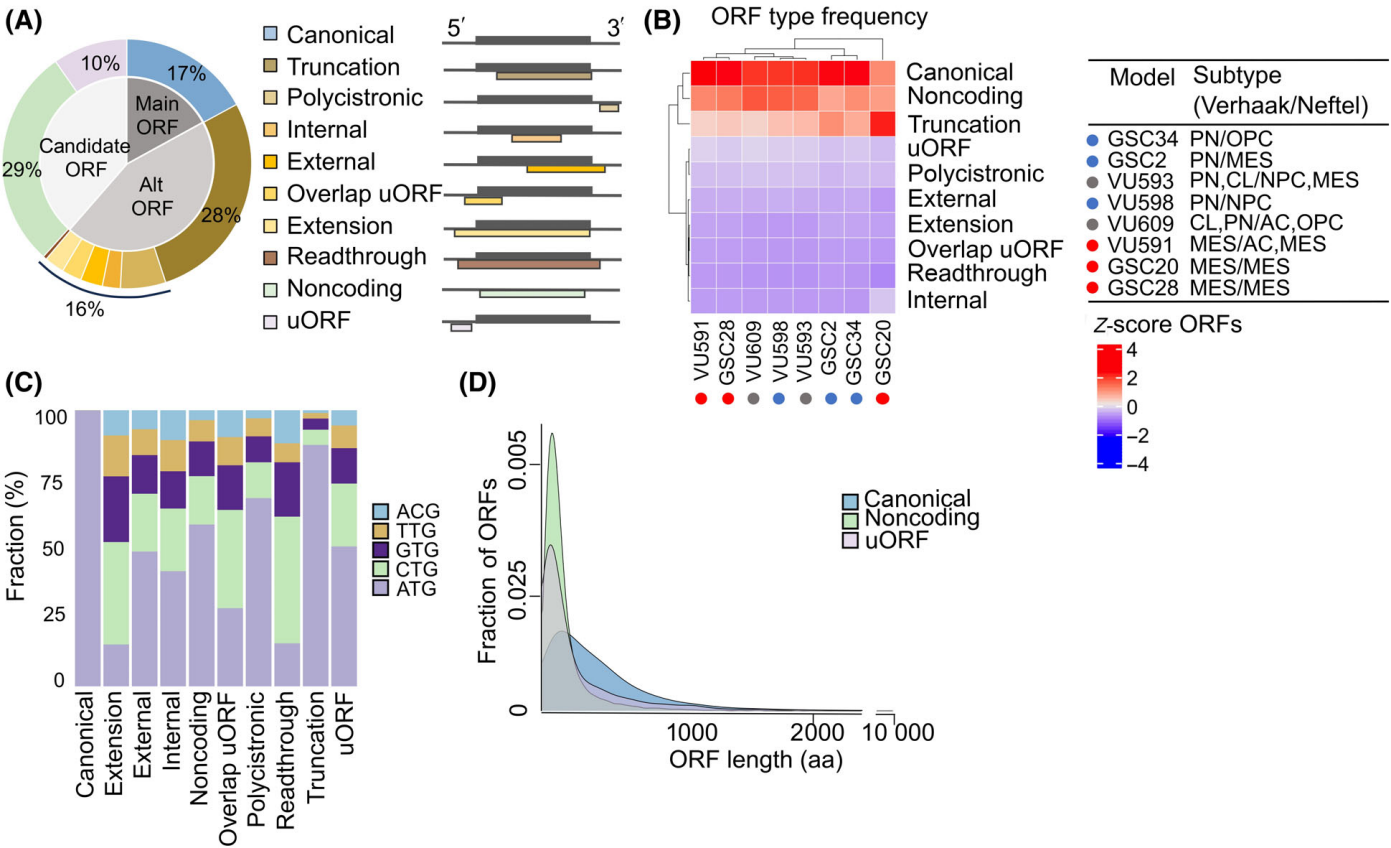
GSCs contain numerous open reading frame types and start codons. (A) Percentage of identified unique ORF types in all GSC samples (*n* = 24, i.e., three different conditions per GSC sample of eight GSC samples in total, based on one technical replicate per sample). Conditions per GSC sample are control (*t* = 0), 30 min after 2 Gy radiotherapy (*t* = 1) and 60 min after 2 Gy radiotherapy (*t* = 2), respectively. Of the identified ORFs 17% are previously annotated (main ORF), 44% are variants of CDSs (alt ORFs), and 39% are candidate ORFs. ORF, open reading frame; uORF, upstream ORF. (B) Correlation of ORF type abundance per GSC sample (*n* = 8, control *t* = 0) and between GSC samples. The relative distribution of ORF types is consistent between GSCs, with canonical, noncoding, and truncation ORFs being most prevalent. There is no relevant association in ORF type abundance per GB subtype. Subtypes are defined and color‐coded in both TCGA transcriptome analysis [64] and single‐cell RNA‐sequencing analysis [42]. AC, astrocyte‐like; CL, classical; MES, mesenchymal; NPC, neural‐progenitor‐like; OPC, oligodendrocyte‐progenitor‐like; PN, proneural. (C) Start codon types per ORF type, wherein canonical ORFs mainly use AUG whereas other ORF types use a variety of start codons. (D) ORF length (amino acids, aa) per ORF type. Overall, candidate ORFs contain shorter ORF lengths (non‐coding median length 312 aa; uORFs median length 318 aa) compared with canonical (median length 912 aa).

Open reading frames (ORFs, i.e., a coding sequence of a gene) are sections that have the potential to be translated into proteins. There is increasing evidence that the protein‐coding capacity of current gene annotations and reference genomes is underreported, motivated by an increasing number of “unannotated proteins” discovered and characterized [24, 25, 49, 50, 51], as well as by the development of Ribo‐seq [52]. In our ribosome profiled GSCs, we discovered both annotated and unannotated ORFs. The ribosome footprints (RPFs) selected by ribosome sequencing can be categorized in ORF classes with RibORF [37]. Across all samples, a total of 259 018 unique ORFs are discovered. These ORFs are located in a total of 81 300 unique genes found in our data. The ORFs can be classified into 10 different categories (Fig. 2A). We categorized ORF types in three main classes; around 17% of the identified ORFs belong to the *main‐ORFs*, referring to annotated and known coding genes (i.e., canonical ORF regions, shown in dark gray). The major fraction of ORFs (44%) was matched to the class of alternative ORFs (*alt‐ORFs*, shown in light gray) derived from alternative start codons in the canonical ORF region. The third major class consisted of genes assumed to be non‐translated and these contain *candidate ORFs* (39%; 33 950 genes, shown in lightest gray), hypothetically representing a class of ncRNAs with coding potential. The translated, assumed non‐coding genes are found in 29% for non‐coding ORFs and 10% for upstream open reading frames (uORFs).

To validate the candidate ORFs (uORF and non‐coding ORFs, respectively), we compared several assigned RibORF parameters using the canonical ORFs as a benchmark, since they are by definition protein‐coding. Indeed, the three‐nucleotide periodicity movement (with the highest fraction of reads in the first nucleotide, e.g., P‐site) characteristic for active translation by ribosomes for candidate ORFs matched with the pattern for canonical ORFs (Fig. S2A). Also, the percentage of maximum entropy (PME) scores measuring the uniformity of read distribution along the ORFs (0 represents highly localized and 1 represents a completely even distribution of reads across codons), showed a comparable PME density distribution compared with canonical ORFs (Fig. S2B). Finally, the predicted translated probability showed similar scores for candidate and canonical ORFs (Fig. S2C). Together, these results indicate that the identified candidate ORFs are undergoing active translation.

Next, we searched for inter‐GSC differences in the ratios between different ORF types. These data indicated that the relative distribution was consistent between the samples (Fig. 2B). The canonical, non‐coding, and truncation ORFs are the most prominent ORF types present throughout the samples. GSC20 was most distinct from the others, with truncated ORFs as the most prominent ORF present. There was no relevant correlation between ORF types and GB subtypes.

Although it was assumed that translation in eukaryotes always initiates at AUG start codons, improvement of techniques with ribosome profiling [23, 32] have revealed thousands of novel initiation events at non‐AUG start codons. The RibORF algorithm considers five types of start codons, respectively ATG, CTG, GTG, TTG, and ACG. In GSCs, all five start codon types for translation initiation were frequently found (Fig. 2C). As expected, canonical ORFs contain mainly AUG start codons (with an extremely small fraction ACG), whereas other ORF types use a variety of start codons. In addition, research mapping genome‐wide translation initiation sites has argued that translation upstream from the canonical region (e.g., overlap uORF, extension, readthrough, uORF) is frequently initiated from non‐AUG start codons [23], most prominently at CTG sites. Indeed, our data confirm this notion (Fig. 2C). Although the CTG start was used in all types (shown in light green), except canonical, they were particularly found in ORFs upstream from the canonical region as well as in non‐coding ORFs (Fig. 2C). Together, these data show that the initiation of translation in GSCs can start at different codons besides the classical AUG for canonical ORFs. The relative use of these new start codons is linked to the type of ORF.

Open‐reading frame lengths of mRNAs are variable in organisms. The length of an ORF impacts the complexity and functionality of the protein it encodes. Longer ORFs generally have a higher likelihood of encoding functional proteins with specific biological roles. Shorter ORFs may encode regulatory peptides or non‐coding RNAs that play essential roles in fundamental biological processes, such as metabolism [53], cell death [54], and development [55, 56]. When comparing the length of candidate ORFs versus canonical ORFs, we found that the identified candidate ORFs contained considerably shorter ORFs (shORFs; median length uORFs 318 amino acids (aa); non‐coding ORFs 312 aa), whereas protein‐coding ORFs were longer (mean length 912 aa; Fig. 2D). Several studies revealed that shORFs can code for bioactive functional peptides [50, 57, 58]. The length of these newly identified shORFs might reflect differences in their functionality when compared to canonical ORFs. As more and more short polypeptides have been identified over the years, functional and mechanistic exploration becomes essential. Knowledge of their role and intracellular behavior will form a new layer of regulation of the proteome and could offer new therapeutic opportunities.

### 3.3 Extensive translation of non‐protein coding biotypes in GB

Accumulating knowledge and evidence support that a substantial fraction of ncRNAs in eukaryotes are functional [59, 60]. However, only a small proportion has yet to reveal biological function [25]. Interestingly, several studies have found that a large fraction of lncRNAs are associated with ribosomes [23, 25, 61].

**Fig. 3:**
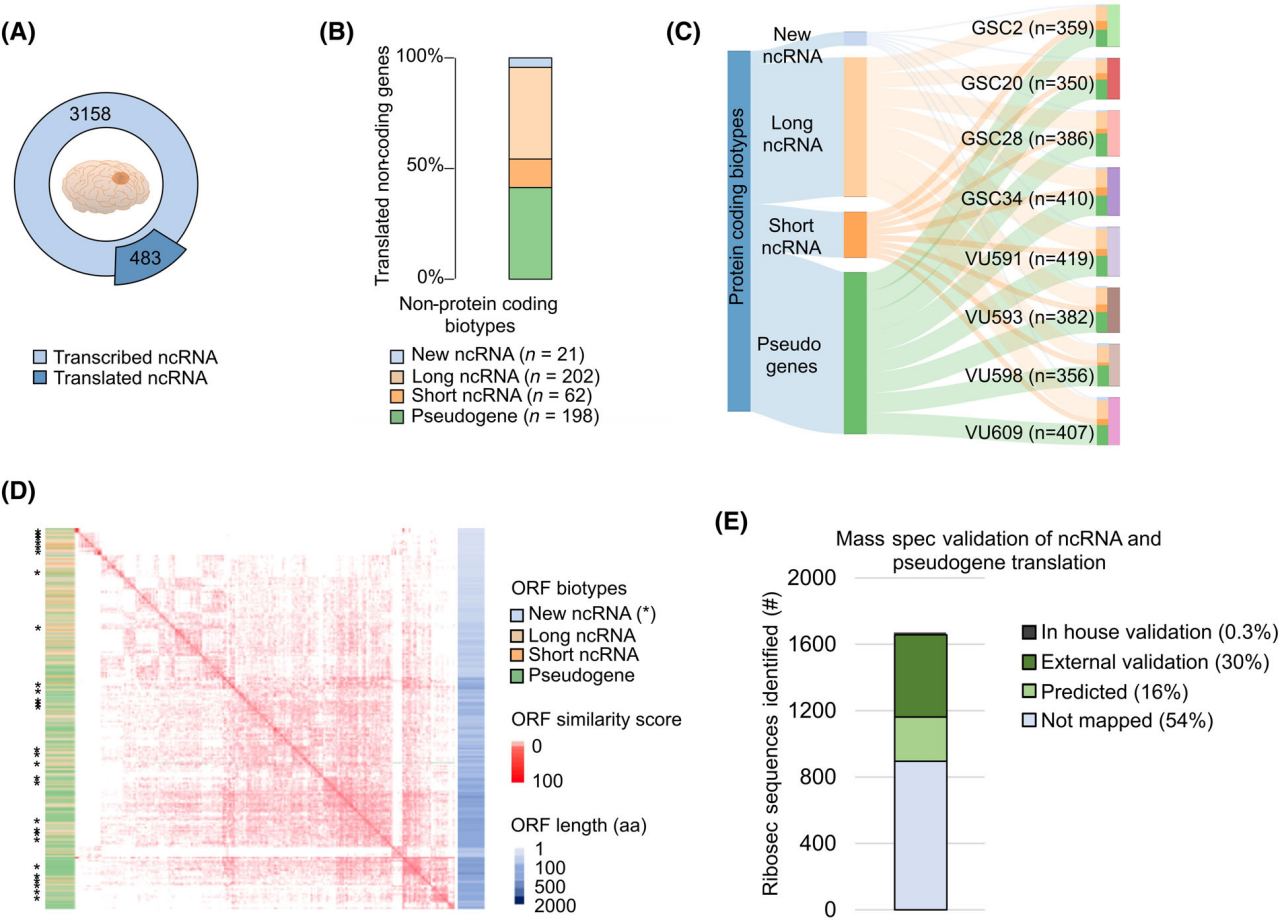
Ribosome profiling reveals non‐protein coding biotypes being translated into GB. (A) Donut chart with the number of non‐coding genes transcribed (*n* = 3158) and translated in the control GSC samples (*n* = 8). About 15% of the transcribed non‐coding genes are being translated (*n* = 483). ncRNA, non‐coding RNA. (B) Translated non‐protein coding biotypes in GSCs, respectively pseudogenes (*n* = 198), short ncRNAs (*n* = 62), lncRNAs (*n* = 202) and newly identified ncRNAs (*n* = 21). (C) Non‐protein coding biotypes presented per patient. As illustrated, the non‐protein biotypes contain the same inter‐patient distribution patterns. (D) Multiple sequence alignment of all identified novel ncRNAs on amino acid level shows that ORFs are aligned based on size (i.e. complexity, depicted in shades of blue) rather than on amino acid similarity (depicted in shades of red). Asterisk (*) in the figure represents where the novel ncRNAs are located in the plot. (E) Histogram showing the validation of the translation of ncRNAs by MS analysis based on our own data as well as data from the Human PeptideAtlas database. Around 30% of the riboseq identified ncRNAs could be validated by external data.

To examine the translation capability of ncRNAs in GSCs, we selected genes with high confidence scores and relatively high expression (mean TPM > 1) in RNA‐seq and Ribo‐seq data across control samples. Of 3158 transcribed non‐coding genes in GSCs, 483 genes were actively being translated (15%, Fig. 3A). Of these translated non‐coding genes, the majority were lncRNAs (*n* = 202, 42%; Fig. 3B) as well as pseudogenes (*n* = 198, 41%), and a smaller group of short ncRNAs (*n* = 62, 13%). Overall, the largest groups were pseudogenes and lncRNAs, which corresponds to data previously published and can express functional proteins [25, 62]. In addition, using our newly developed bioinformatic method we discovered 21 new ncRNAs that are plausibly undergoing translation (Fig. 3B). We found that the distribution of the translated non‐protein coding biotypes was overall evenly across all samples, indicating that the proportions of the potential expressed biotypes was equivalent between GB cell lines (Fig. 3C). By characterizing the expression patterns and functional roles of translated ncRNA types in GB, we can gain mechanistic insights into the molecular processes underlying tumor development and progression. This knowledge may lead to the identification of targeted therapies to improve patients' outcomes.

We next extracted the amino acid sequences of all identified ORFs of the potentially translated non‐protein coding biotypes from ENSEMBL [63]. To investigate conservation on the amino acid level of non‐coding ORFs, we used multiple sequence alignment (MSA, clustalw). Overall, although some clustering of ORFs encoded by pseudogenes and lncRNAs was found (Fig. 3D, Table S1), the distribution based on size (depicted in shades of blue) and class suggests a lack of strong conservation, that is, clustering reflects sequence complexity rather than conservation. In agreement, most alignments showed pairwise matches of alternative ORFs from the same gene.

To confirm that the newly identified non‐coding RNAs are expressed at the RNA level, we performed qPCR on two cell lines (VU598 and GSC34, respectively). After compensating for putative genomic DNA contamination, this showed that a substantial amount of the identified non‐coding RNAs are expressed at the RNA level (Fig. S3, primers are shown in Table S1). RNA expression was also validated on external single‐cell RNAseq data (Fig. S4A–D), showing that a substantial number of non‐coding RNAs are expressed in cancer cells as well as in other normal cells present in the tumor (i.e., macrophages, oligodendrocytes, and T‐cells).

To validate the translation of non‐coding RNAs, we used in‐house generated as well as external MS data, available through the Human PeptideAtlas [41]. This validation showed that up to 29% of the riboseq‐identified peptides could be confirmed by MS (see Table S1 for an overview and Table S2 for the actual mapping). We also performed MS on four GSC cell lines which confirmed the translation of four ncRNAs (see Table S2). A summary of the MS validation is shown in Fig. 3E.

To investigate the therapeutic relevance of the riboseq‐identified ncRNAs, we analyzed the dependency on these RNAs in GB using DepMap RNAi data models [45]. This enables us to identify the lethal effects upon knockdown of the respective non‐coding RNAs in GB (representative of *n* = 58 genes in *n* = 65 cell lines). Lethal phenotypes are assessed through a gene dependency score, where a negative value is reflective of viability loss after the knockdown of the respective target gene (Fig. S5, Table S1). This showed that a minority of the ncRNA genes showed a lethal phenotype, in particular for the genes PI4KAP1; TXLNGY; ARHGAP27P1‐BPTFP1‐KPNA2P3; SBDSP1; FOXO3B, and HERC2P3. It is important to recognize that conclusions drawn from DepMap RNAi models, which are based on classical glioma cell lines, may be limited in their interpretive value since they might not accurately represent the tumors observed in clinical settings. Therefore, further functional validation of the identified ncRNAs is necessary in future research.

Taken together, our results show that GB contains non‐protein encoding biotypes that are actively being translated. The function of the genes remains to be elucidated and requires further deeper analysis. The translation of non‐protein coding biotypes in cancer suggests a previously overlooked layer of functional complexity. Understanding these translated products may unveil novel cancer mechanisms and biomarkers, broadening our understanding and improving cancer diagnosis and treatment strategies.

### 3.4 The translational landscape of GB matches transcriptional subtypes and ontology differences in extracellular properties and cell adhesion

**Fig. 4:**
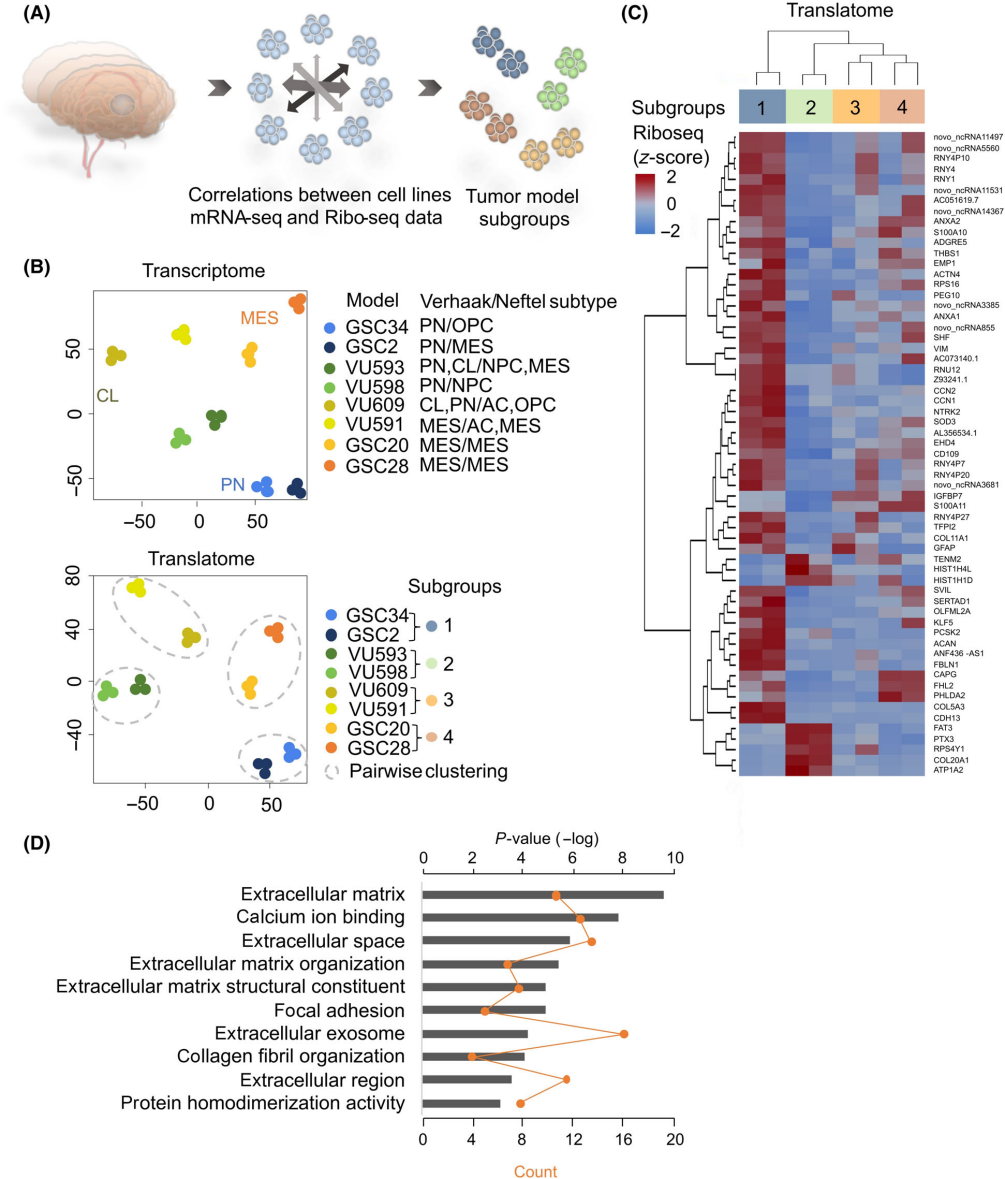
GSCs can be classified into four subgroups based on gene expression on translational and transcriptional levels, matching previously published GB subtypes. (A) Schematic overview of the analysis, eight patients at *t* = 0 (control) were correlated by unsupervised clustering to identify subgroups on transcriptional and translational level. (B) Dimension reduction analysis using tSNE of the transcriptome (mRNA‐seq, upper panel) and translatome (Ribo‐seq, lower panel) of the GSCs resulted in four subgroups (subgroup 1 GSC2, GSC34; subgroup 2 VU593, VU598; subgroup 3 VU609, VU591; subgroup 4 GSC20, GSC28, respectively). Correlation of the patients on both transcriptional and translational levels resulted in similar subgroups. Subgroups 1 and 4 were classified respectively as the Proneural (PN) and Mesenchymal subtypes, according to previously published TCGA GB subtype data [64] (Fig. S6A). Classification of subgroups based on public data using the TCGA transcriptome analysis [64] or single‐cell RNA‐sequencing analysis [42] is shown for each GSC cell line. AC, astrocyte‐like; CL, classical; MES, mesenchymal; MES, mesenchymal‐like; OPC, oligodendrocyte‐progenitor‐like; PN, proneural. (C) Corresponding heatmap of 61 differentially expressed (DE) genes at *t* = 0 (control) at translatome level between subgroups. (D) Top 10 GO terms (DAVID functional analysis) matching the DE genes of C, matching subgroup 1 (PN). The orange dots show the number of genes that are common between the GO term's gene set and the respective DE gene set. The gray bars are the −log of the *P*‐value determined by DAVID functional analysis (*P*‐value < 0.0001).

Molecular characteristics of gene expression based on transcriptome data by the Cancer Genome Atlas Program (TCGA) [64] have resulted in the definition of distinct GB subtypes, respectively, Mesenchymal (MES), Proneural (PN), and Classical (CL) [64, 65, 66]. Recently, single‐cell RNA‐sequencing (scRNA‐seq) [42] revealed a limited set of cellular states of GB malignant cells that recapitulate distinct neural cell types, respectively, neural‐progenitor‐like (NPC‐like), oligodendrocyte‐progenitor‐like (OPC‐like; TCGA‐PN), astrocyte‐like (AC‐like; TCGA‐CL), and mesenchymal‐like (MES‐like; TCGA‐MES) states [42]. Here, we aimed to identify subgroups between our cell lines on both transcriptome and translatome levels and searched for genes differentially expressed between the subgroups (Fig. 4A). To identify cell line subtypes, we performed gene signature analysis using both transcriptome [64] (Fig. S6A,B) and scRNA‐seq [42] (Fig. S6C) signatures. As shown, cell lines showed distinct (i.e., GSC20, GSC28 both MES subtypes) to more mixed phenotypes (i.e., GSC34, VU593, VU598, VU609 containing elements of PN and CL) and phenotypes were less clearly defined for GSC2 and VU591.

We visualized cell line differences on transcriptome and translatome levels using tSNE dimensionality reduction. We observed a similar pattern between GBs transcriptome and translatome (Fig. 4B). Subgroups based on transcriptome data matched translatome data, indicating that overall patterns at the transcriptome level were also present at the translatome level. These data show that the subtype classification of GB seems maintained at translatome levels, although this conclusion is based on limited sample numbers and *in vitro* models.

We classified the cell lines into four subgroups, based on our tSNE dimension reduction analysis (i.e., GSC34/GSC2 (a), VU593/VU598 (b), VU609/VU591 (c), and GSC20/GSC28 (d); Fig. 4B). Next, we performed differential expression (DE) analysis of the subgroups on both translatome and transcriptome level. At the translatome level, a total of 61 genes (*P* adj < 0.01) are differentially expressed, with subgroups 1 and 2 being most distinct from each other (Fig. 4C, Fig. S6D; DE genes are depicted in Table S1). Interestingly, DAVID gene‐ontology (GO) analysis [67, 68] of the DE genes showed predominant associations with extracellular matrix (ECM) organization and cell adhesion between subgroups, acting as a dynamic interface between cells and their surroundings and providing structural support (Fig. 4D). Overall, these genes were expressed at higher abundance in the PN group (GSC2, GSC34) when compared with the other subgroups. For example, protein actinin alpha 4 (ACTN4), is involved in the crosslinking of actin filaments thereby affecting F‐actin dynamics and migration ability of cancer cells [69]. In addition, ACTN4 is associated with poorer overall survival in GB patients [70], rendering it a potential target for therapeutic intervention. Another DE gene is SH2 domain‐containing adapter protein F (SHF), expressed in the PN group (Fig. 4C, Table S1). This protein is found to be a tumor suppressor in GB, by negatively regulating STAT3 activity that upon activation promotes self‐renewal and prevents apoptosis [71, 72]. Downregulation of SHF predicts poor outcomes in GB, making SHF/STAT3 interaction an interesting therapeutic target [71].

We performed a similar DE analysis for the transcriptome data with the cell line subgroups previously mentioned (Fig. 4B). We identified 650 DE genes (*P*‐value < 0.0001, Fig. S6E,F; DE genes are depicted in Table S1). GO analysis resulted in comparable results with the translatome analysis, showing GO term enrichment for cell adhesion, ECM and ECM organization and extracellular activity of ion channels and receptors, suggesting the importance of extracellular properties and GBs microenvironment on GBs phenotype. An example of a DE gene is Dysadherin (FXYD4), a cell membrane glycoprotein involved in modulating cell–cell adhesion, whereof its expression level is correlating with metastatic behavior in for example breast cancer and head and neck cancer [73, 74, 75], expressed at higher abundance in subgroups 2 and 3 and lower abundance in subgroup 1 (i.e., PN group) of our analysis. Cell adhesion molecule 2 (CADM2) is more expressed in subgroup 1, involved in maintenance of cell adhesion and tumor suppression, inhibiting glioma proliferation, migration, and invasion [76] (Fig. S6G). To conclude, subgroups based on transcriptome data matched translatome data. In our data, subgrouping is mainly based upon DE genes involved in ECM organization, serving as a crucial mediator of cellular responses to environmental cues, of which the composition and mechanical properties can be altered in response to changes in the cellular environment. Targeting the ECM organization represents a promising path for developing novel therapeutic interventions.

### 3.5 Radiotherapy effects are correlated with histone dynamics

**Fig. 5:**
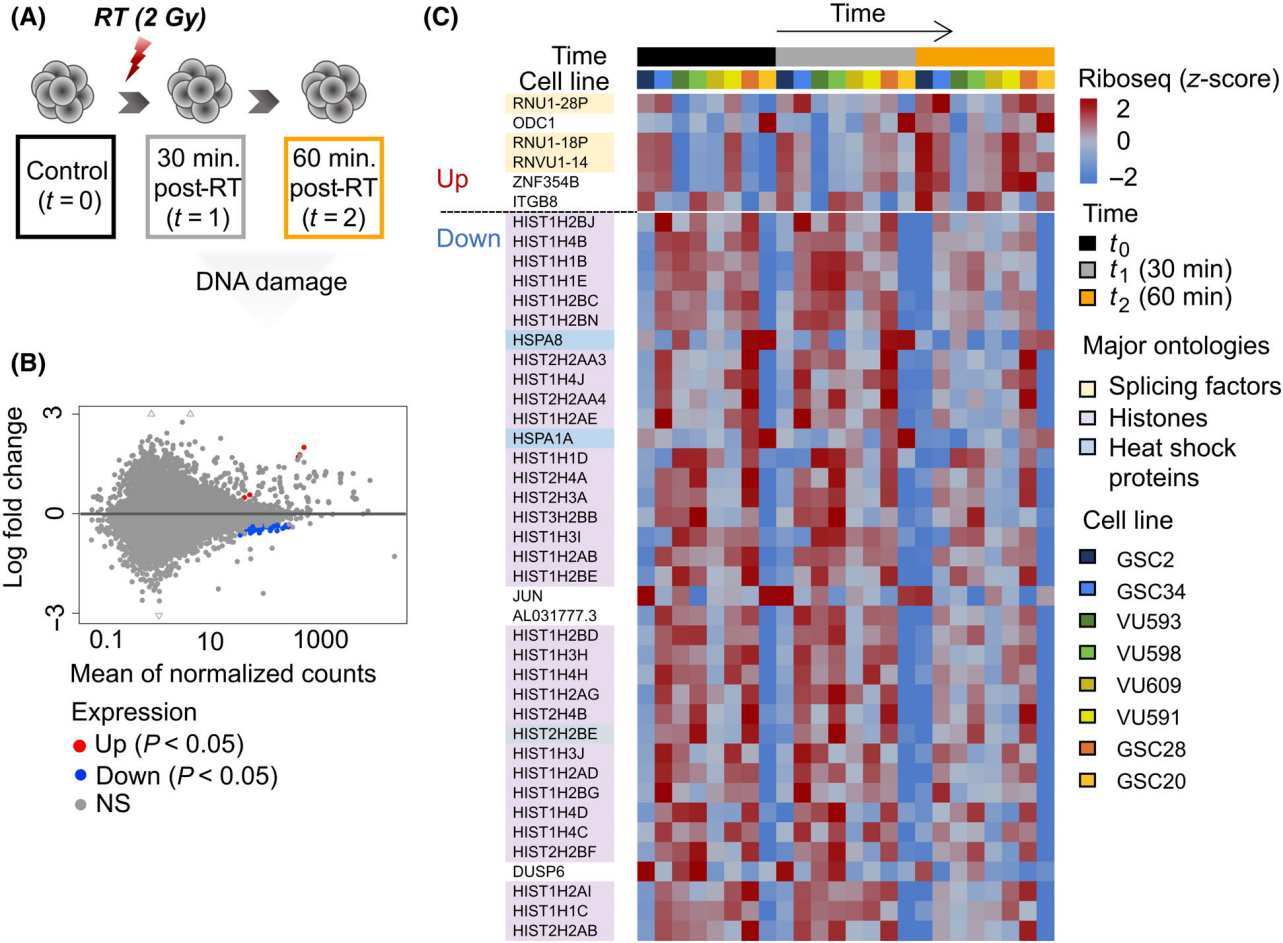
Direct effect of radiotherapy results in alterations of histone and splicing factor dynamics in GB. (A) Schematic overview of the experiment, we used eight GSC cell lines in total and were radiated once with 2 Gy. Ribosome profiling and mRNA sequencing was performed on respectively control (*t* = 0), 30 min after irradiation (*t* = 1) and 60 min after irradiation (*t* = 2). Gy, gray; RT, radiotherapy. (B) MA plot illustrating 43 genes were significantly differentially expressed over time after radiation, (adjusted *P*‐value < 0.05), respectively, six genes up‐ and 37 downregulated (NS, non‐significant; *P*, adjusted *P*‐value). *P*‐values were determined by Wald's test. (C) Corresponding heatmap of 43 DE genes over time, wherein mainly histone genes are lower expressed after irradiation and splicing factors are higher expressed over time. Gy, gray; NS, non‐significant; RT, radiotherapy; *t*_0_, control; *t*_1_, 30 min after radiotherapy; *t*_2_, 60 min after radiotherapy.

Clinical exposure to irradiation is a successful strategy although radioresistance commonly occurs and results in activation of the DNA damage checkpoint response and increased levels of DNA repair [6]. Radiotherapy creates double‐strand breaks (DSBs) in DNA. DSB repair is linked to rapid changes in epigenetic modifications, including histone methylation resulting in the recruitment of DNA repair proteins that can change the local chromatin structure, hereby impacting chromatin accessibility and gene expression patterns (i.e., epigenetic modifications) [77, 78, 79]. Interestingly, many GB tumors have significant epigenetic dysregulation driving its pathophysiology and therapy resistance [80]. For example, increased histone acetylation has been observed following radiotherapy, which is associated with enhanced transcriptional activation of DNA repair genes. Conversely, radiation‐induced DNA damage can also lead to histone hypoacetylation and changes in histone methylation patterns, affecting chromatin compaction and gene silencing. To determine the dynamics of GBs translatome under therapeutic pressure, we studied cells when exposed to irradiation, to measure the direct effects of radiotherapy on translation. Cells were irradiated at three different timepoints, respectively 30 and 60 min after radiation and control before radiation. Cells were exposed once with 2 Gy, the same quantity a GB patient would receive daily [81]. In parallel, we determined radiation sensitivity in a time series experiment of 6 cell lines (GSC2, GSC20, GSC28, GSC34, VU591, and VU593, respectively), by calculating the relative effect by dividing the slope of the growth curve of spheroid controls by the growth curve of spheroids after 2 Gy radiation for three consecutive days (Fig. S7A,B) [36]. Reduction of growth was seen in all cell lines, and reduced proliferation was observed at around 5 days after radiotherapy. Note that none of the GSC cultures is completely resistant to radiotherapy. To study the direct effect of radiotherapy on translation, GSCs were irradiated once with 2 Gy and lysed 30 min (*t* = 1) and 60 min (*t* = 2) after radiation (Fig. 5A). deseq analysis on Ribo‐seq data revealed, respectively, six higher expressed and 37 lower expressed genes (*P‐*adj < 0.05) after radiotherapy (Fig. 5B), wherein histone genes and heat shock proteins are mostly affected by radiation (Fig. 5C). According to the literature, several of the differentially expressed genes we identified have already been studied and associated with radiosensitivity in cancer. For example, the expression of HSPA1A contributes to radioresistance in breast cancer [82] and DUSP6 regulates radiosensitivity in GB [83] and esophageal squamous cell carcinoma [84]. Furthermore, ITGB8 is associated with radiotherapy in glioma patients [85] and inhibition of ITGB8 leads to radio sensitization of GB cells through postmitotic cell death [86].

Among the DE genes with increased translation levels are pseudogenes or variants of U1 snRNA [87, 88], involved in splicing through an RNA mediated mechanism. In general, the crosstalk between histone activity and splicing factors plays a critical role in regulating gene expression, chromatin structure, and cellular functions. The functional consequence of the increased translation of the splicing factors that function on the RNA level is as yet unknown. Histone genes and heat shock proteins show reduced translation levels after radiotherapy (Fig. 5C). When we ranked the cell lines based on radiosensitivity, we noticed that the relative radiosensitive cell lines have a strongest change in the translation of histones, as compared to the least radiosensitive cell lines (Fig. S7C). Due to the low number of cell lines we cannot draw any hard conclusions, but there appears to be a correlation between histone translation after radiotherapy and radiotherapy‐sensitivity (*r* = 0.623 at *t*_2_ = 60 min, Fig. S7D). The reduced translation of histones after radiotherapy might result in a decrease in histone proteins and histone activity, altering the chromatin structure which possibly affects the accessibility of DNA to transcription factors and other regulatory proteins, ultimately influencing gene expression patterns. Proteins that are downregulated are by definition redundant for targeted therapy. Conversely, targeting the chromatin remodeling complex that is altered by histone expression levels might be of interest in GB. The SWI/SNF complex is a multi‐subunit complex regulating gene expression, DNA repair, and development [89]. Recent research has shown that so‐called bromodomain (i.e., proteins that recognize acetylated lysine residues on N‐terminal tails of histones) inhibitors of the SWI/SNF complex enhance DNA damage and cell death in GB, sensitizing GB to Temozolomide and Bleomycin [90]. Bleomycin is radiomimetic, causing single and double‐strand breaks in DNA similar to that of ionizing radiation [91]. Consequently, bromodomain inhibitors might be an interesting drug in combination with radiotherapy, indirectly modulating histone dynamics and gene expression in GB. Furthermore, the enhanced efficacy of histone deacetylase inhibitors (HDACs) combined with bromodomain inhibitors have synergistic efficacy against GB cells [92], making the combination of bromodomain inhibitors with radiotherapy an interesting strategy for future research. Together, our results show the impact of radiotherapy on histone translation, underscoring the intricate interplay between DNA damage response mechanisms and epigenetic regulation, which ultimately determines the effectiveness of radiotherapy treatment. Further research is needed to understand the impact of radiotherapy on the crosstalk between histones and splicing factors influencing chromatin accessibility in GB, elucidating the molecular mechanisms underlying treatment response and resistance to improve patient outcomes.

## 4 Discussion

In this study, we investigated the translatome of GB models. This analysis identified up to 10‐fold more genes compared to mass spectrometry, providing a broader perspective on the GB translatome. We also found that previously published subtypes based on RNA expression are reflected on the translation level. In addition, the technique revealed a broad spectrum of open reading frame types in both coding and non‐coding regions, where, remarkably, a set of lncRNAs and pseudogenes were found to be translated and up to 29% of the respective peptides could be confirmed by in‐house and external MS data. A technical innovation of our study lies in the ribosome profiling analysis in combination with a novel bioinformatic analysis method, which enabled the identification of new mRNAs and their corresponding ORFs, which would remain hidden by previously used strategies where these genes would remain unannotated. We have confirmed the transcription of a substantial number of potential new translated ncRNAs by qPCR. Finally, we show that the translation of histones and splicing factors is highly affected after irradiation of GSCs and their protein abundance is correlated with radiosensitivity, providing new insights into immediate intracellular dynamics under radiotherapeutic conditions.

### 4.1 Alternative protein isoforms and the choice of non‐AUG start codons

Profiling of translation initiation has revealed that many genes have extended or truncated products, starting either at AUG or non‐AUG codons [23, 93, 94, 95]. However, the functional significance of these protein variants in eukaryotes is largely unexplored. Here, we show that GSCs contain numerous ORF types using alternate translation initiation sites with a large quantity of truncated ORFs, that could cause changes in protein function. A known example from the literature is a truncated form of the antiviral protein MAVS, that by leaky scanning results in a “truncated” protein isoform (miniMAVS) that inhibits the signal transduction pathway of MAVS in macrophages [95]. This finding and additional evidence suggests that eukaryotes may commonly translate more than one protein from a single mRNA, commonly associated with bacterial or viral translation, than previously thought [23, 96].

From the ORF types found in our study, many, except canonical ORFs, use multiple start codons for the initiation of translation. Yet, it is shown that usage of non‐AUG start codons changes during the development of cells and upon stress. For instance, translation of MRPL18 (encoding a mitochondrial ribosomal subunit protein) during heat shock results in alternative translation initiation from a CTG site downstream of the commonly used AUG start codon [97]. This protein variant is incorporated into cytoplasmic rather than mitochondrial ribosomes, regulating the increased synthesis of HSP70 protein during heat shock. In this case, the change in the start codon orchestrates translation initiation that impacts cell survival.

Also, non‐AUG translation is associated with multiple human diseases, including cancer progression and neurodegeneration [98, 99, 100, 101]. Yet, how the selection of non‐AUG codons is regulated is still poorly understood, nevertheless, dysregulation of eukaryotic initiation factors (eIFs, i.e., protein complexes involved in the initiation phase of eukaryotic translation) has been observed in several pathologies including cancer [102, 103, 104]. Dysregulated eIFs cause overall initiation precision of ORFs and start codon choice [99]. Recently, it was shown that eIF2A promotes the translation of non‐AUG uORFs in squamous cell carcinomas (SCC), positively regulating their downstream oncogenic ORFs by modulating the scanning mechanism of the ribosome or altering the fidelity of start codon recognition [100]. In addition, deleting eIF2A in SCCs resulted in the reduction of malignant progression [100]. Overall, in GB multiple ORF types exist, resulting in many alternative protein isoforms. These are mainly translated via non‐AUG start codons, also seen in other cancer types and during cellular stress response, driving cancer proliferation. The selection of non‐AUG initiation start sites in cancer cells is a complex process regulated by multiple factors, including initiation factors and genetic or epigenetic alterations. To what extent this is tumor‐specific in the case of GB still needs to be confirmed by analyzing normal tissues.

### 4.2 Non‐coding RNAs as a potential source of new peptides

A large number of cancer‐related lncRNAs have been discovered based on cancer cell transcriptome profiling [105], whereof a couple of functional lncRNA peptides have been described in various cell types [25, 55, 106, 107]. We validated the translation of ncRNAs by using the Human PeptideAtlas database. This led to the identification of a substantial number of peptides that are synthesized from lncRNAs and pseudogenes (*n* = 501). In‐house confirmation of translated peptides by MS proved to be challenging since the few overlapping peptides (*n* = 5) identified by MS are also part of larger open reading frames, which cannot rule out that these are degradation products of full‐length versions of the open reading frames. Recent ribosome profiling studies have also found non‐protein coding biotypes (e.g., pseudogenes and lncRNAs) being associated with ribosomes [22, 25]. Moreover, numerous proteomics studies have found that many human pseudogenes and lncRNAs produce peptides [25, 107, 108, 109, 110, 111]. Another possibility is that these translated ncRNAs/lncRNAs are both coding and non‐coding. Such double roles have been described previously and are known to exist for several lncRNAs [25, 112, 113, 114].

Our homology analysis showed a limited level of similarity which indicates that lncRNA or pseudogene‐encoded peptides could have a wide array of functions. They could function as components of protein complexes, as signal molecules, as translation regulators or act as ribosome sequestration units. DepMap analysis demonstrating the essential role of ncRNAs, showed that some of the identified genes showed a weak lethal phenotype in GB cell lines. Moreover, several studies indicate that the ncRNAs we identified in our translatome data do have functional roles in cancer. For instance, overexpression of circRPPH1_025 promotes migration and invasion of glioblastoma [115]. In addition, IGF2BP2 induces chemoresistance in glioblastoma cells [116] and lncRNA GAS5 represses gliomas stemness and malignancy [117]. LINC00511 and lncRNA SNHG20 contribute to glioblastoma tumorigenesis [118, 119]. Both lncRNA SOX2‐OT and SNHG6 promote glioma progression and cell growth [120, 121] and lncRNA DLEU promotes cell proliferation in glioblastoma [122, 123]. We conclude in general that we have identified many lncRNAs that are translated into peptides and our data provide a resource for further technical validation as well as to investigate their biological role.

In this study, a couple of (novo) ncRNA‐encoded genes were identified and we confirmed the transcription of their respective RNAs by qPCR, although we have not individually proven their translation. Independent confirmation remains technically challenging not only because of the small size of the peptides, which interferes with mass‐spec or click‐it labeling detection, but also because of other experimental challenges that should be resolved in future studies. To find new ncRNAs that are potentially coding with our in‐laboratory developed Ribo‐seq bioinformatic method is encouraging for the GB research field, providing an expansion of GB translatome and mechanistic insights. However, since there is a significant interpatient and intrapatient expression diversity within GB, it seems very challenging to find druggable molecules shared by a larger group of GB patients. Yet, the potential new ncRNAs identified need further deeper analyses to investigate their function in cells.

### 4.3 Histones are less active translated immediately after radiation

In GB, radioresistance is a major cause of treatment failure and recurrence [5, 6]. However, there is not much knowledge about the factors related to radiotherapy resistance in GB. In this study we show that irradiation induces decreased histone translation. Recently, it was shown that chronic irradiation of human cells reduces histone levels, on both mRNA and protein level, and deregulates gene expression through chromatin decompaction and elevated RNA synthesis leading to cell senescence [124]. Phosphorylation of gamma‐H2AX plays an important role in DNA repair in GSCs after irradiation [36]. We did not see a downregulation of translation 1 h after irradiation of gamma‐H2AX in our data indicating that a new equilibrium of histone isotypes might arise after irradiation, although intranuclear localization and functional studies are required to reveal this. Also, histone modifications after radiotherapy may play a role in genome destabilization and cellular senescence. Dysregulated histone translation may contribute to radioresistance by promoting DNA repair and cell survival pathways.

Pseudogenes/variants of U1 snRNA, which guide 5′ splicing site selection by the spliceosome through RNA‐based base pairing [125], were found to be translated in our study and this translation was increased by irradiation. Since splicing regulation relies on the RNA nature of the U1 snRNA itself, it is unlikely that translation of this RNA species plays a role in splicing regulation *per se*. Since splicing regulates the repair of double‐strand breaks in DNA after irradiation in GB [126, 127], the increased translation could reflect a higher abundancy or availability of U1 snRNA. Recently, increased translation of splicing factors was also found in GB cell lines after irradiation [128].

Together, decreased translation of histones as a result of radiotherapy was found as a consistent factor in our analysis for multiple GSCs, which could affect cell proliferation and highlight that histone translation could be a novel target for GB therapy.

## 5 Conclusion

Our comprehensive study of the translatome landscape in GB models has unveiled valuable insights into the complex protein translation mechanisms that contribute to GBs behavior. We identified 10 times more genes being actively translated with ribosome profiling compared to MS, which offers a more comprehensive view of GBs translatome. Our findings indicate that previously established GB subtypes based on transcriptome data are similar on the translational level. Furthermore, a wide range of ORFs are discovered within coding and non‐coding regions. Notably, we discovered a set of lncRNAs and pseudogenes that were translated, with up to 29% of the associated peptides confirmed by in‐house and external MS data. A key innovation of our research is the ribosome profiling analysis coupled to a novel bioinformatics approach, allowing us to identify new mRNAs and their corresponding ORFs that would have remained unannotated using traditional methods. Lastly, our results demonstrate that the translation of histones and splicing factors is significantly impacted following irradiation of GSCs, thereby providing new insights into the immediate intracellular dynamics under radiotherapeutic conditions. Further exploration and functional validation of these differentially expressed genes could provide pivotal mechanistic insights essential for improving outcomes in patients battling this aggressive form of cancer. This work enriches the existing data resources for GB research but also lays the groundwork for future investigations into translationally regulated genes and their potential as biomarkers for diagnosis and treatment strategies.

## Conflict of interest

The authors declare no conflict of interest.

## Author contributions

CM, BAW, and FMGC developed the conceptual framework and FMGC conducted the ribosome profiling and RNA sequencing laboratory experiments. ZH developed the Ribosome profiling analysis code for new non‐coding RNAs. EC performed independent validation experiments. ZH and FMGC performed the bioinformatic analyses. RRH conducted the mass spectrometry laboratory work and SRP performed the bioinformatic analyses on mass spectrometry. AB‐C performed qPCRs and qPCR analyses. CM, BAW, CRJ, WPV, and DN enabled the project by providing conceptual ideas, resources, and supervision. FMGC and BAW wrote the paper. All authors reviewed the paper.

### Peer review

The peer review history for this article is available at https://www.webofscience.com/api/gateway/wos/peer‐review/10.1002/1878‐0261.13743.

## Supporting information


**Fig. S1.** Transcripts per million of mRNA and Ribo‐seq data.
**Fig. S2.** Read densities of ribosome profiling data.
**Fig. S3.** Expression data of ncRNAs found with ribosome profiling that are potentially coding, confirmed by qPCR in GSC34 and VU598.
**Fig. S4.** Non‐coding RNA expression using single cell RNAseq data. Non‐coding RNAs identified by ribosome profiling were analyzed for their expression in the single‐cell clusters of normal cells as well as tumor cells.
**Fig. S5.** Lethal effect of riboseq‐identified ncRNAs.
**Fig. S6.** Subgroup DE analysis on transcriptome and translatome level.
**Fig. S7.** Radiation sensitivity GSCs.

**Table S1.** Supporting information on mRNA and ribo‐seq data.

**Table S2.** (novo)ncRNA‐encoded identified peptides in GB.

## Data Availability

All used scripts are available at: https://github.com/Arthurhe/RiboReadCountByFrame. All presented Ribo‐seq data are available at GEO under accession number GSE229866 (https://www.ncbi.nlm.nih.gov/geo).
