# Supplementary material for: The translatome of glioblastoma
Source: Mol Oncol. 2024 Oct 17;19(3):716–40. doi: 10.1002/1878-0261.13743 (PMC11887679; doi:10.1002/1878-0261.13743)
Supplement: Supplementary file 1 — Fig. S1. Transcripts per million of mRNA and Ribo‐seq data. Fig. S2. Read densities of ribosome profiling data. Fig. S3. Expression data of ncRNAs found with ribosome profiling that are potentially coding, confirmed by qPCR in GSC34 and VU598. Fig. S4. Non‐coding RNA expression using single cell RNAseq data. Non‐coding RNAs identified by ribosome profiling were analyzed for their expression in the single‐cell clusters of normal cells as well as tumor cells. Fig. S5. Lethal effect of riboseq‐identified ncRNAs. Fig. S6. Subgroup DE analysis on transcriptome and translatome level. Fig. S7. Radiation sensitivity GSCs. [file MOL2-19-716-s002.zip › Figure_legends.docx]

**Supplementary figure legends**

**Supplementary Figure 1**

**Transcripts per million of mRNA and Ribo-seq data**

1. Histogram of mRNA-seq and Ribo-seq data of all samples combined (24 total; 8 GSC cell lines with 3 time points per cell line). Genes with mean tpm>1 are depicted in yellow. tpm, transcripts per million.
2. Histogram of Ribo-seq data of all samples combined (24 total; 8 GSC cell lines with 3 time points per cell line). Genes with mean tpm>1 are depicted in yellow. tpm, transcripts per million.

**Supplementary Figure 2**

**Read densities of ribosome profiling data**

1. Fractions of reads in 1st, 2nd and 3rd nucleotides (nt) of indicated open reading frame (ORF) types for respectively canonical, non-coding and uORFs (upstream ORFs). Help lines in dotted grey represent the fraction of reads in the first nucleotide of codons (left figure), which are high. The reads in the second (middle figure) and third (right figure) are low, typical for the 3-nucleotide movement of a ribosome translating mRNA.
2. Corresponding percentage of maximum entropy (PME) values for indicated ORF types (0 represents highly localized and 1 represents a completely even distribution of reads across codons).
3. Corresponding predicted p-values for indicated ORF types. A cutoff of 0.7 was used (false positive rate 0.0095, true positive rate 0.936) to select ORFs.

**Supplementary Figure 3**

**Expression data of ncRNAs found with ribosome profiling that are potentially coding, confirmed by qPCR in GSC34 (A) and VU598 (B).** Each qPCR reaction corresponds to approximately 25 ng RNA that was either transcribed into cDNA (+RT) or not treated to assess genomic DNA contamination (-RT). Note that two independent primer sets were used for each target except for the housekeeping genes TBP and ActB (shown in pink). Three technical replicates were performed per primer set. The number of asterisks above each gene corresponds to the level of significance (*<0.05, **<0.01, ***<0.001, ****<0.0001, all Bonferroni corrected because of multiple testing). In case primer dimers were observed, a cycle threshold of 40 was taken so as not to overestimate the level of significance.

**Supplementary Figure 4**

**Non-coding RNA expression using single cell RNAseq data. Non-coding RNAs identified by ribosome-profiling were analyzed for their expression in the single-cell clusters of normal cells as well as tumor cells.**

1. Scatterplot showing the expression of ncRNAs in single-cell RNAseq data of Neftel *et al.* 2019 (n=7,930 cells). Cellular subgroups were based on DBSCAN selection (Epsilon: 5.95 and Min pts: 80). Normal cells could easily be identified because these formed cellular clusters that were shared between patients. This was seen for T-cells (identified by CD8A expression), macrophages and microglia (identified by CD68/CD168 expression) as well as oligodendrocytes (identified by OLIG2 expression). The remaining cells showed individual clusters representative of single patients and were considered pure tumor cells.
2. Dotplot showing the expression of lncRNAs in cancer cells as well as normal cells including T-cells, macrophages and microglia as well as oligodendrocytes.
3. Dotplot showing the expression of pseudogenes in the different cell populations.
4. Dotplot showing the expression of short ncRNAs in the different cell populations.

**Supplementary Figure 5**

**Lethal effect of riboseq-identified ncRNAs**

Some riboseq-identified non-coding RNAs (ncRNAs) show a lethal effect upon their knockdown in GB cell lines (for n=58 in n=65 cell lines). Data were taken from the RNAi Achilles+DRIVE+Marcotte, DEMETER2 study. Lethal phenotypes are shown as a gene dependency score, where a negative value is reflective of viability loss after the knockdown of the respective target gene. Genes are top-ranked for their average lethal phenotype.

**Supplementary Figure 6**

**Subgroup DE analysis on transcriptome and translatome level**

1. Heatmap of GSCs classified into 4 subgroups based on K-means clustering using genes differentially expressed between three published GB subgroups; respectively proneural, mesenchymal and classical (Verhaak *et al.* 2010; Wang *et al.* 2017). Only untreated samples were used. Expression levels are shown as z-value.
2. Radar plot on the visualizing the average z-score of the MSigDB signatures for the different subtypes according to Verhaak *et al.* 2010. Note: signatures are not necessarily mutually exclusive but rather show mixed phenotypes. K-means clustering and heatmap-platting of the left figure were executed using the online bioinformatic tool R2 (version 3.3.4; [www.https://hgserver1.amc.nl/](http://www.https://hgserver1.amc.nl/)).
3. Radar plot visualizing the average z-score of MSigDB signatures for the different subtypes according to Neftel *et al.* 2019, after K-means clustering (heatmap not shown).
4. MA plot visualizing differentially expressed genes (blue dots) between subgroups on translatome (Ribo-seq) level (n=61, *p* adj <0.01)
5. MA plot visualizing differentially expressed genes (blue dots) between subgroups on transcriptome (mRNA-seq) level (n=650, *p* adj <0.0001).
6. Differentially expressed genes of transcriptome data between patient subgroups (*p* adj <0.001), respectively subgroup 1 GSC34 and GSC2; subgroup 2 VU598 and VU593; subgroup 3 VU609 and VU591, subgroup 4 GSC28 and GSC20). tSNE dimension reduction revealed similar subgroups on both transcriptional and translational levels (Fig. 4B).
7. Top 19 GO terms (DAVID functional analysis) matching the DE genes of Fig. S6F. The orange dots show the number of genes that are common between the GO term’s gene set and the respective DE gene set. The grey bars are the -log of the p-value determined by DAVID functional analysis (*p*-value <0.0001)*.*

**Supplementary Figure 7**

**Radiation sensitivity GSCs**

1. Dose-response curves of 6 GSC cell lines treated with radiotherapy. Spheroids were irradiated with 2Gy for three consecutive days, from day 0 onwards. Spheroid volume was measured for 21 days in total. The slope of the growth curves is depicted in grey dotted lines per cell line.
2. The relative effect of radiotherapy on spheroid growth was calculated by dividing the control slope by the slope of irradiated cells per cell line, ranked by their relative effect. Radiotherapy led to a relatively reduced spheroid growth in time ranging from 1,4-5,5 compared with control.
3. DE genes after radiotherapy per cell line, with cell lines ranked from most to least sensitive at time points t0 and t2 (60 mins after 2Gy radiotherapy). Sensitive cell lines have a relatively high abundance of histone and heat shock proteins and a low abundance of splicing factors, compared with relatively insensitive cell lines.
4. Scatterplot showing the relation between radiation sensitivity and the relative histone translation after therapy. This shows a correlation between histone translation after therapy and the relative sensitivity to radiation.
