# Supplementary figures and images for: The translatome of glioblastoma
Source: Mol Oncol. 2024 Oct 17;19(3):716–40. doi: 10.1002/1878-0261.13743 (PMC11887679; doi:10.1002/1878-0261.13743)

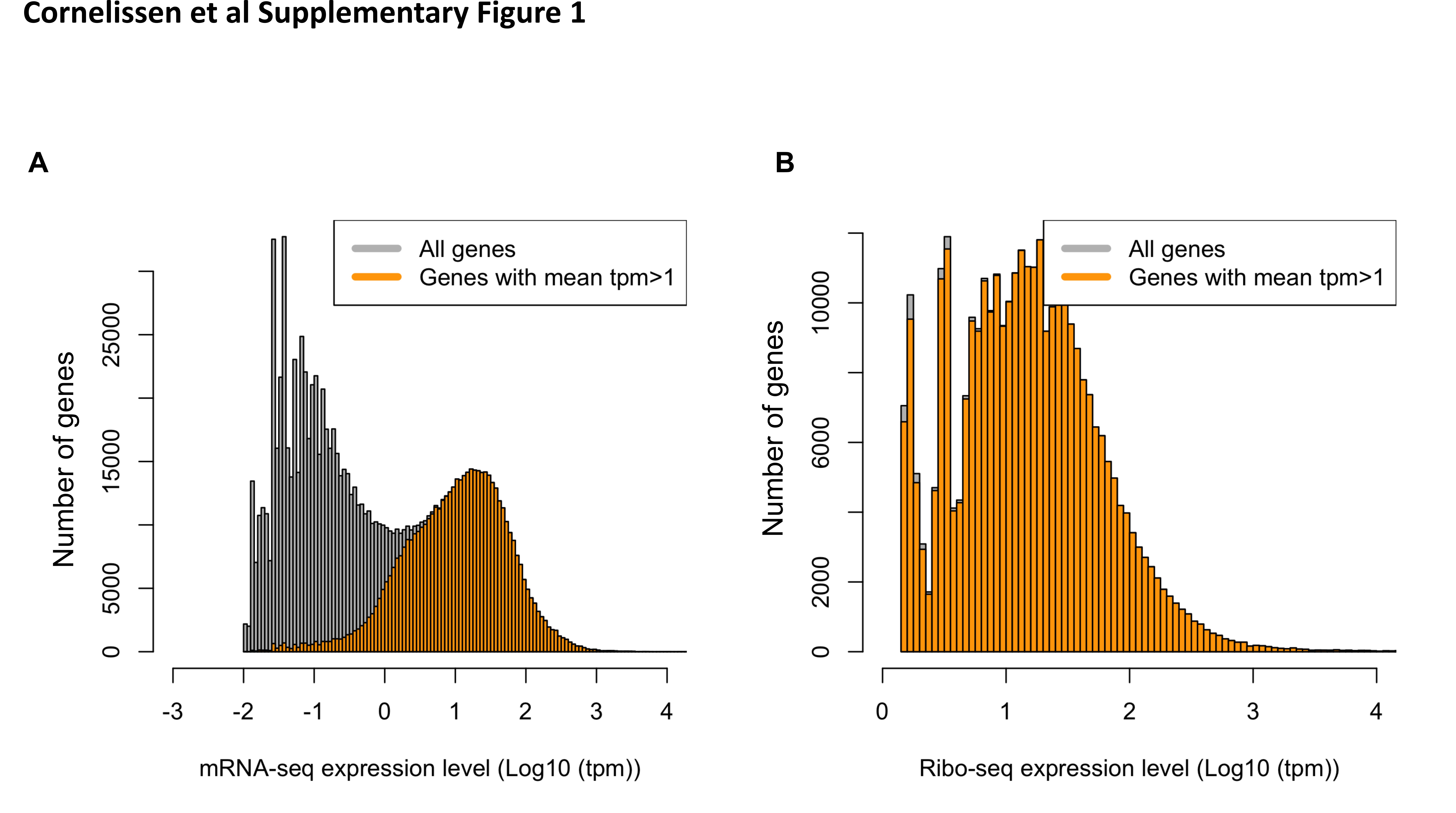

Supplement: Supplementary file 1 — Fig. S1. Transcripts per million of mRNA and Ribo‐seq data. Fig. S2. Read densities of ribosome profiling data. Fig. S3. Expression data of ncRNAs found with ribosome profiling that are potentially coding, confirmed by qPCR in GSC34 and VU598. Fig. S4. Non‐coding RNA expression using single cell RNAseq data. Non‐coding RNAs identified by ribosome profiling were analyzed for their expression in the single‐cell clusters of normal cells as well as tumor cells. Fig. S5. Lethal effect of riboseq‐identified ncRNAs. Fig. S6. Subgroup DE analysis on transcriptome and translatome level. Fig. S7. Radiation sensitivity GSCs. [file MOL2-19-716-s002.zip › FigureS1.jpg]

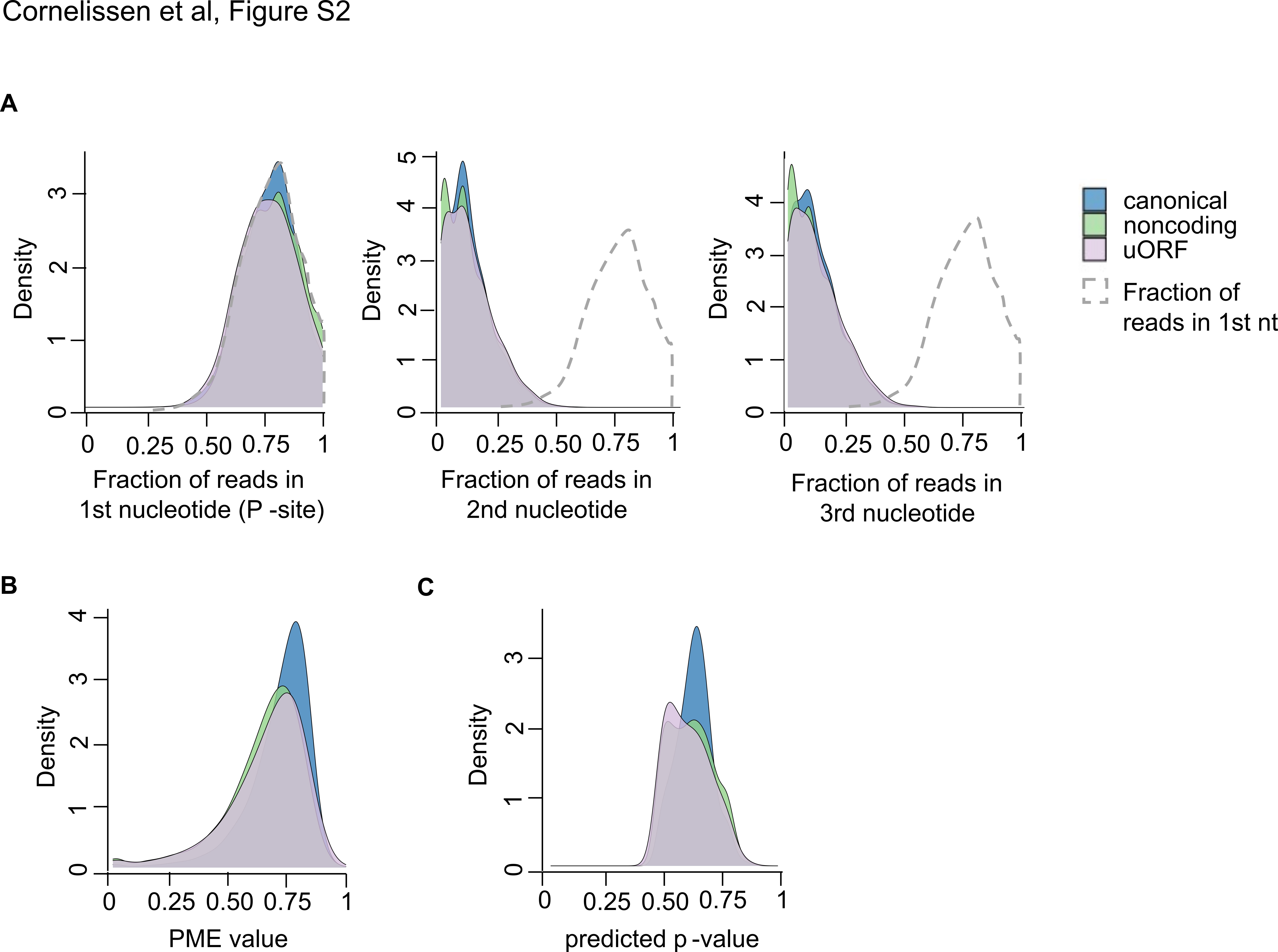

Supplement: Supplementary file 1 — Fig. S1. Transcripts per million of mRNA and Ribo‐seq data. Fig. S2. Read densities of ribosome profiling data. Fig. S3. Expression data of ncRNAs found with ribosome profiling that are potentially coding, confirmed by qPCR in GSC34 and VU598. Fig. S4. Non‐coding RNA expression using single cell RNAseq data. Non‐coding RNAs identified by ribosome profiling were analyzed for their expression in the single‐cell clusters of normal cells as well as tumor cells. Fig. S5. Lethal effect of riboseq‐identified ncRNAs. Fig. S6. Subgroup DE analysis on transcriptome and translatome level. Fig. S7. Radiation sensitivity GSCs. [file MOL2-19-716-s002.zip › FigureS2.jpg]

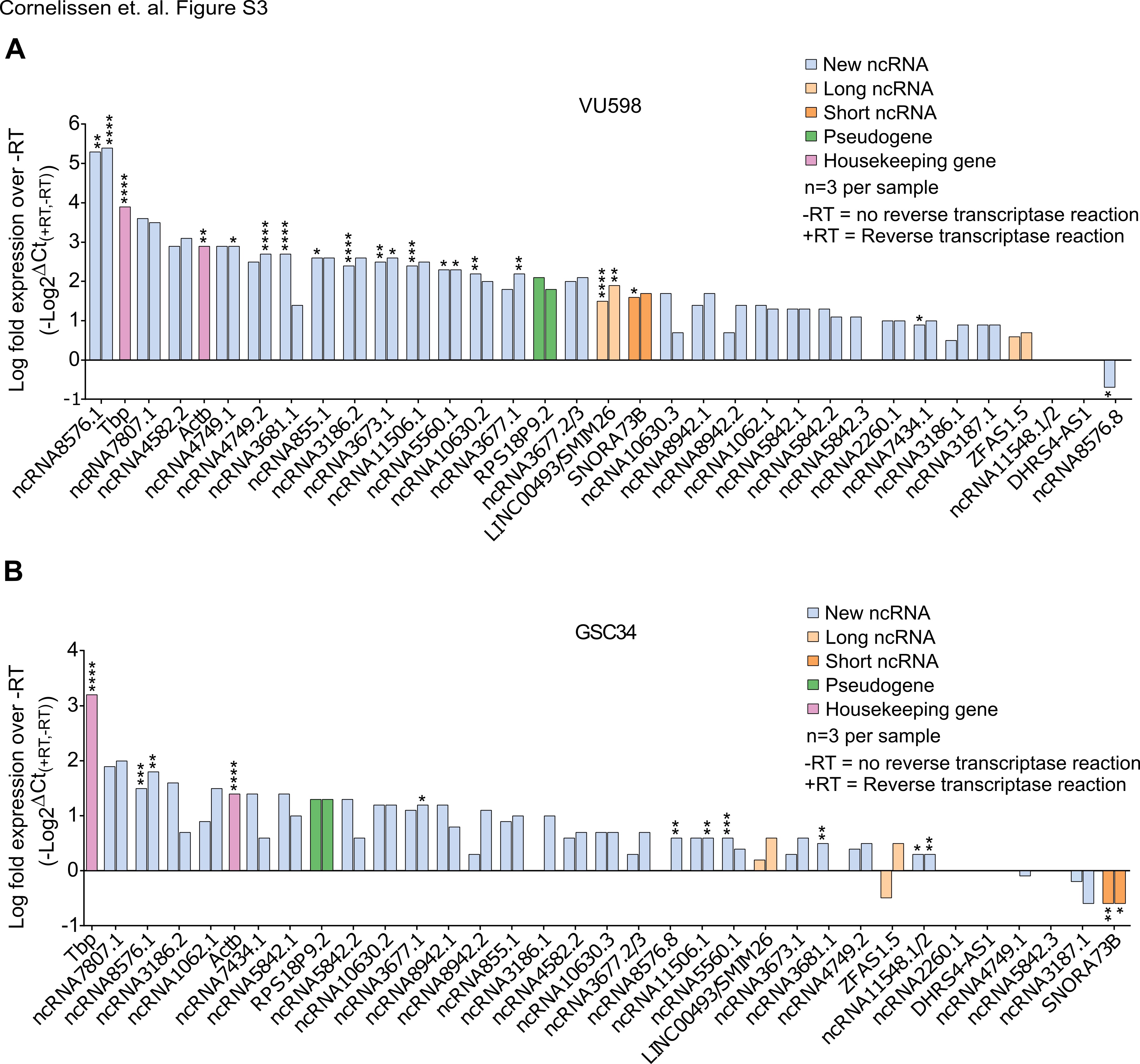

Supplement: Supplementary file 1 — Fig. S1. Transcripts per million of mRNA and Ribo‐seq data. Fig. S2. Read densities of ribosome profiling data. Fig. S3. Expression data of ncRNAs found with ribosome profiling that are potentially coding, confirmed by qPCR in GSC34 and VU598. Fig. S4. Non‐coding RNA expression using single cell RNAseq data. Non‐coding RNAs identified by ribosome profiling were analyzed for their expression in the single‐cell clusters of normal cells as well as tumor cells. Fig. S5. Lethal effect of riboseq‐identified ncRNAs. Fig. S6. Subgroup DE analysis on transcriptome and translatome level. Fig. S7. Radiation sensitivity GSCs. [file MOL2-19-716-s002.zip › FigureS3.jpg]

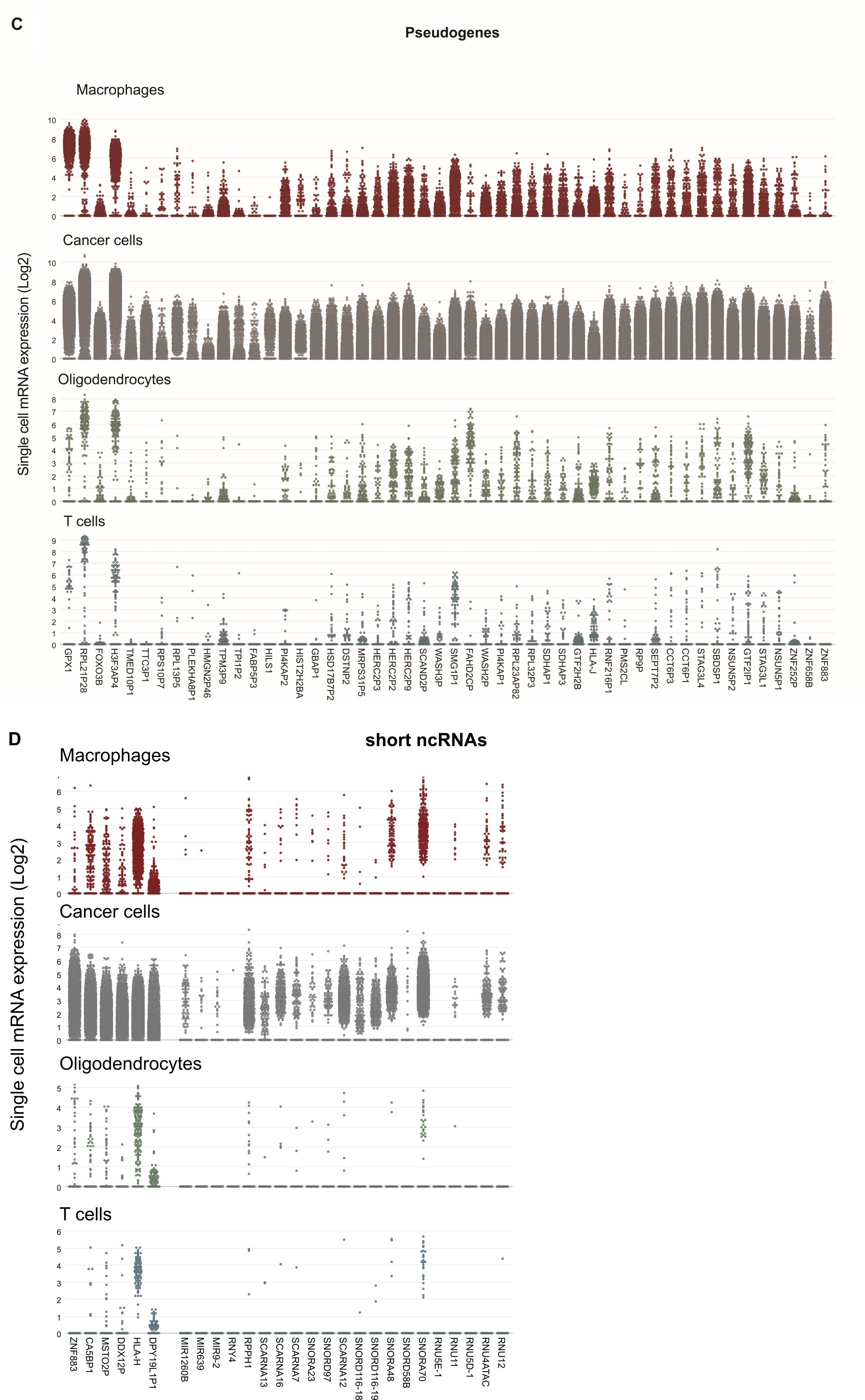

Supplement: Supplementary file 1 — Fig. S1. Transcripts per million of mRNA and Ribo‐seq data. Fig. S2. Read densities of ribosome profiling data. Fig. S3. Expression data of ncRNAs found with ribosome profiling that are potentially coding, confirmed by qPCR in GSC34 and VU598. Fig. S4. Non‐coding RNA expression using single cell RNAseq data. Non‐coding RNAs identified by ribosome profiling were analyzed for their expression in the single‐cell clusters of normal cells as well as tumor cells. Fig. S5. Lethal effect of riboseq‐identified ncRNAs. Fig. S6. Subgroup DE analysis on transcriptome and translatome level. Fig. S7. Radiation sensitivity GSCs. [file MOL2-19-716-s002.zip › FigureS4CD.jpg]

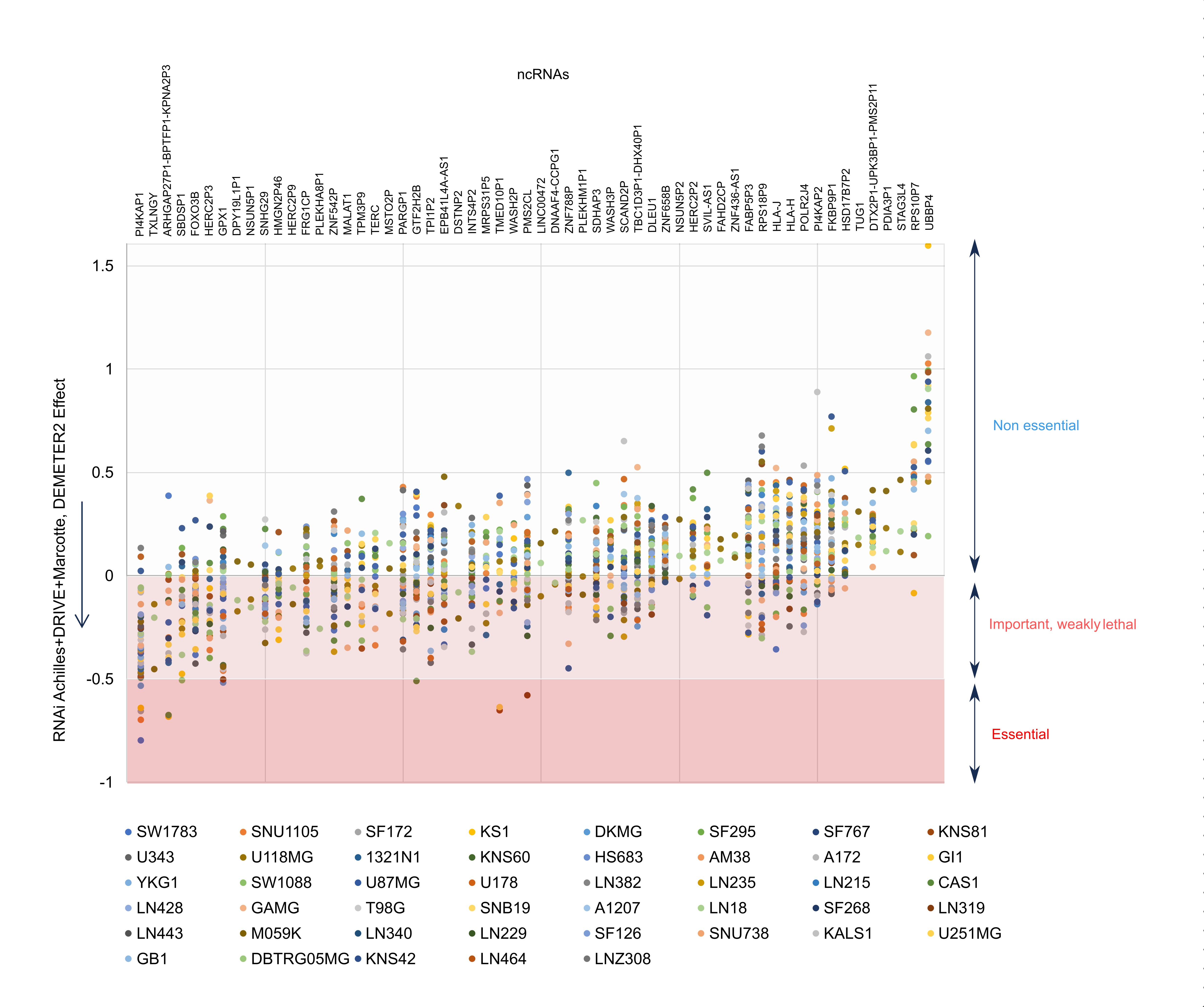

Supplement: Supplementary file 1 — Fig. S1. Transcripts per million of mRNA and Ribo‐seq data. Fig. S2. Read densities of ribosome profiling data. Fig. S3. Expression data of ncRNAs found with ribosome profiling that are potentially coding, confirmed by qPCR in GSC34 and VU598. Fig. S4. Non‐coding RNA expression using single cell RNAseq data. Non‐coding RNAs identified by ribosome profiling were analyzed for their expression in the single‐cell clusters of normal cells as well as tumor cells. Fig. S5. Lethal effect of riboseq‐identified ncRNAs. Fig. S6. Subgroup DE analysis on transcriptome and translatome level. Fig. S7. Radiation sensitivity GSCs. [file MOL2-19-716-s002.zip › FigureS5.jpg]

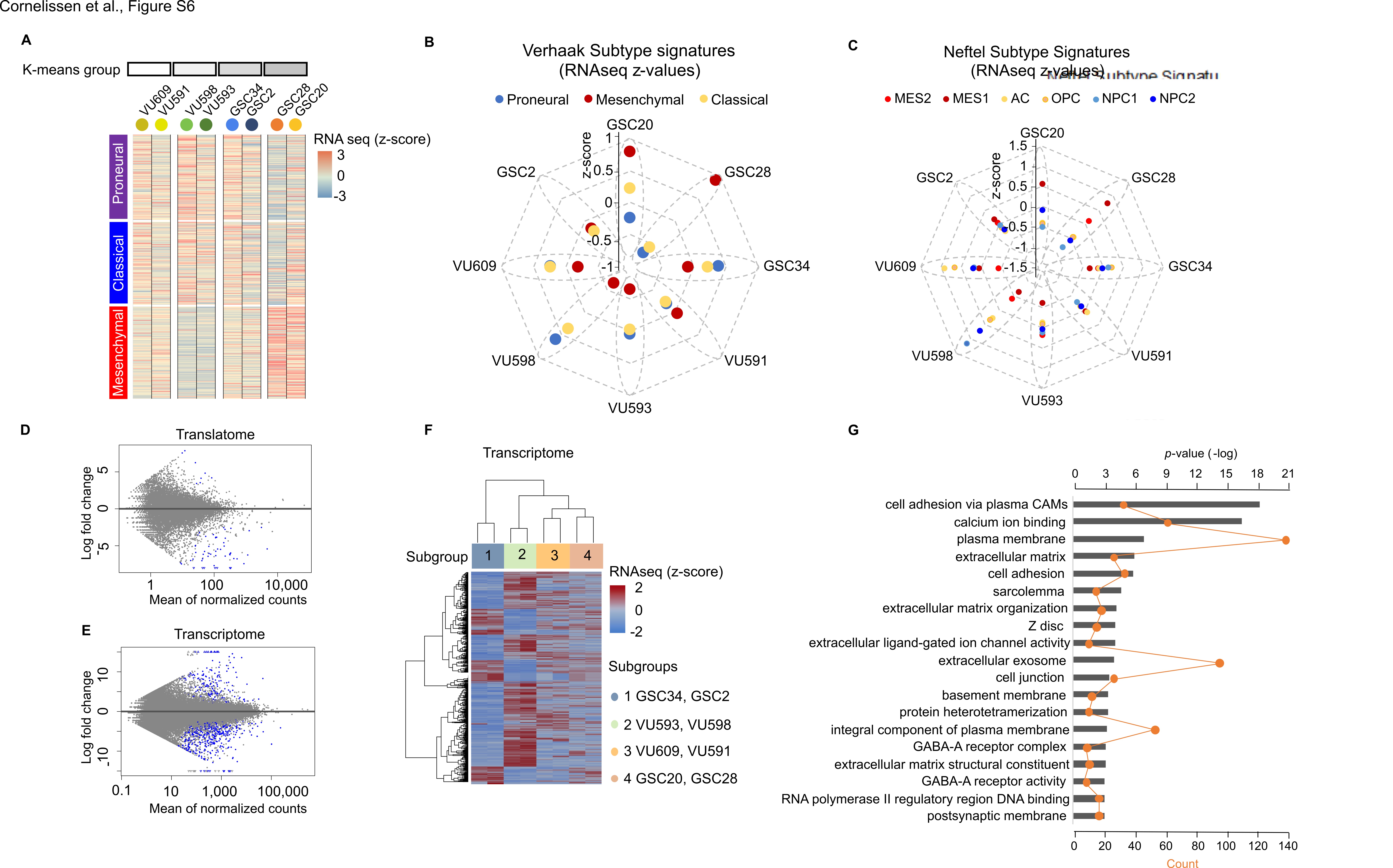

Supplement: Supplementary file 1 — Fig. S1. Transcripts per million of mRNA and Ribo‐seq data. Fig. S2. Read densities of ribosome profiling data. Fig. S3. Expression data of ncRNAs found with ribosome profiling that are potentially coding, confirmed by qPCR in GSC34 and VU598. Fig. S4. Non‐coding RNA expression using single cell RNAseq data. Non‐coding RNAs identified by ribosome profiling were analyzed for their expression in the single‐cell clusters of normal cells as well as tumor cells. Fig. S5. Lethal effect of riboseq‐identified ncRNAs. Fig. S6. Subgroup DE analysis on transcriptome and translatome level. Fig. S7. Radiation sensitivity GSCs. [file MOL2-19-716-s002.zip › FigureS6.jpg]

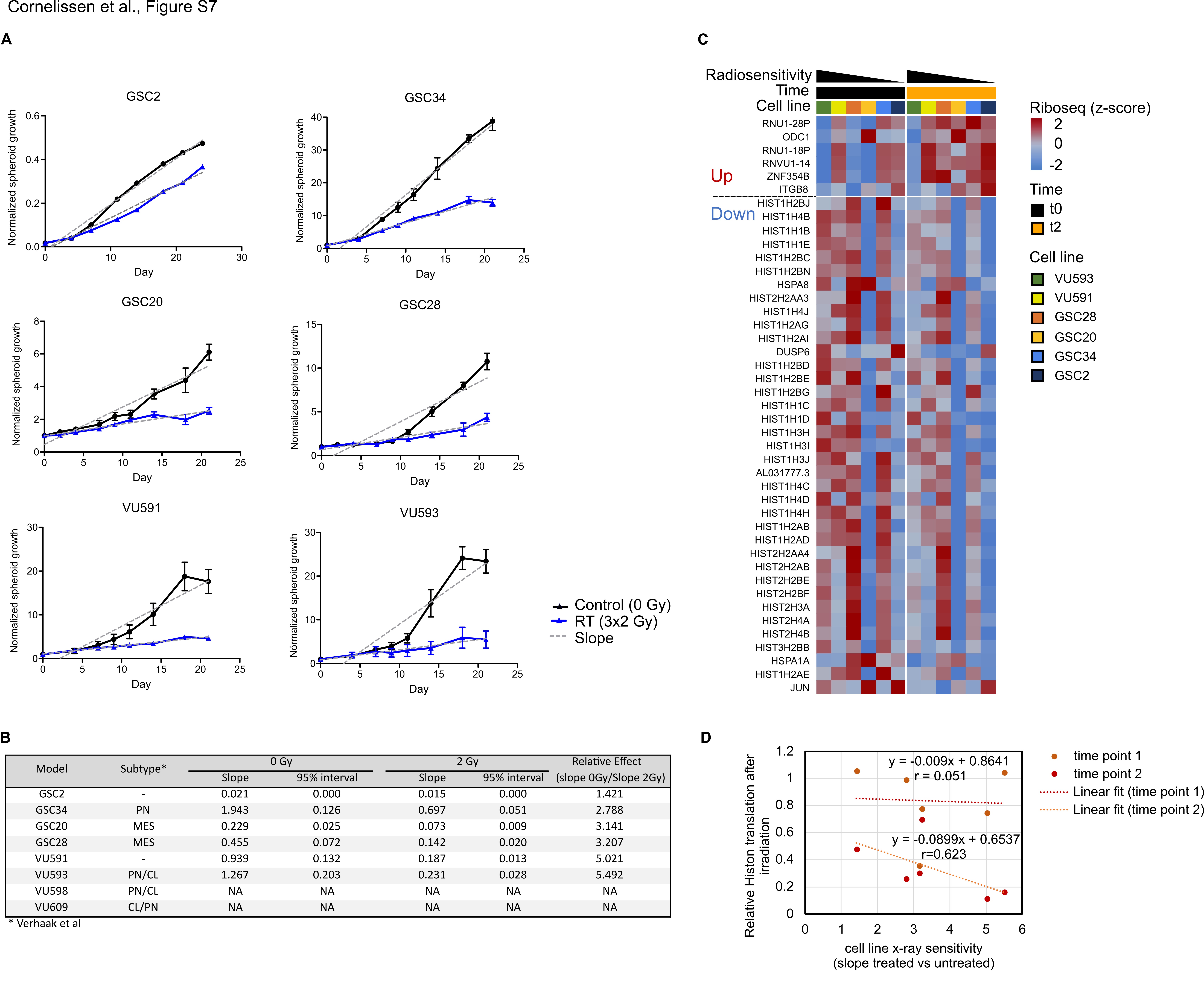

Supplement: Supplementary file 1 — Fig. S1. Transcripts per million of mRNA and Ribo‐seq data. Fig. S2. Read densities of ribosome profiling data. Fig. S3. Expression data of ncRNAs found with ribosome profiling that are potentially coding, confirmed by qPCR in GSC34 and VU598. Fig. S4. Non‐coding RNA expression using single cell RNAseq data. Non‐coding RNAs identified by ribosome profiling were analyzed for their expression in the single‐cell clusters of normal cells as well as tumor cells. Fig. S5. Lethal effect of riboseq‐identified ncRNAs. Fig. S6. Subgroup DE analysis on transcriptome and translatome level. Fig. S7. Radiation sensitivity GSCs. [file MOL2-19-716-s002.zip › FigureS7.jpg]

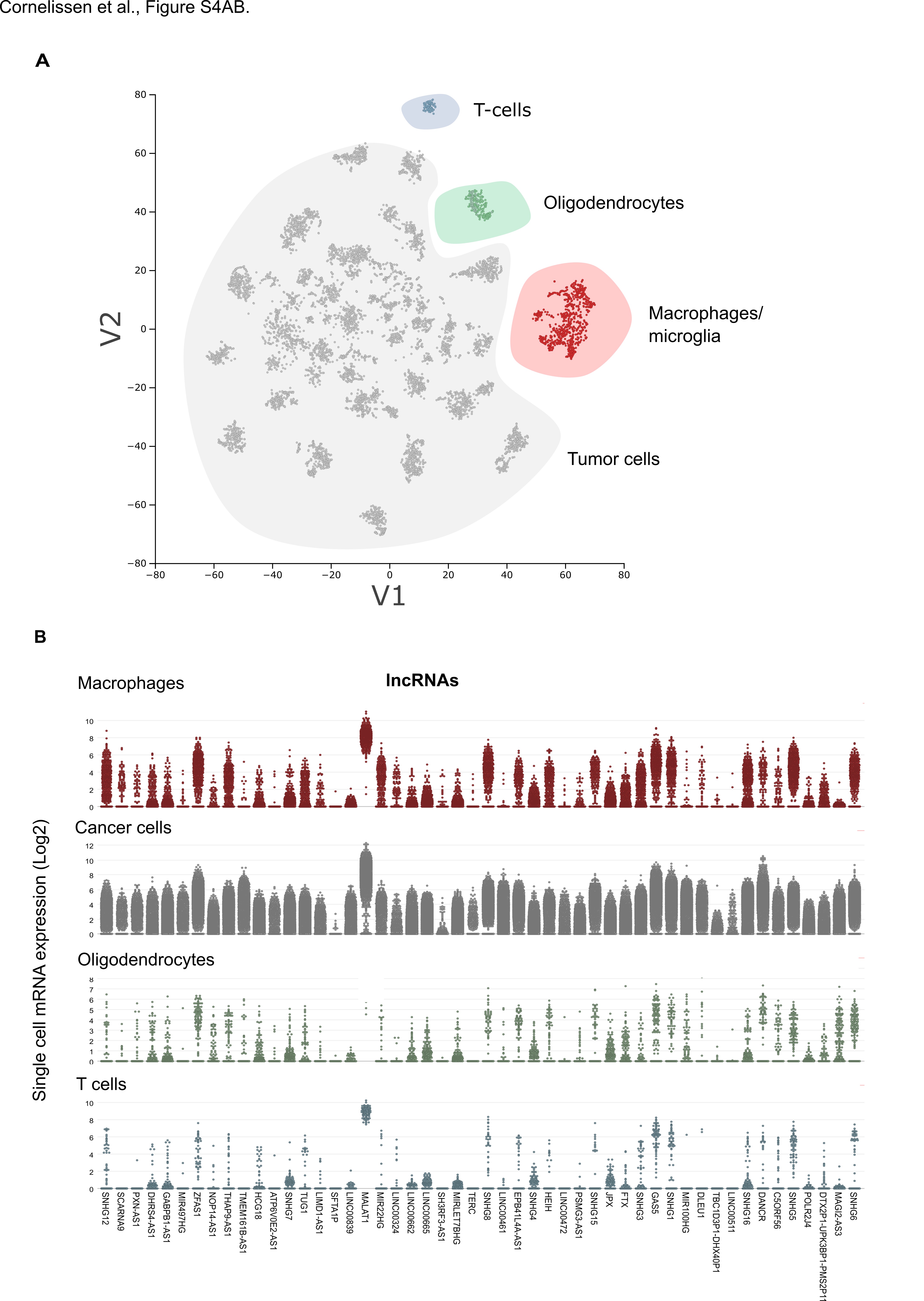

Supplement: Supplementary file 1 — Fig. S1. Transcripts per million of mRNA and Ribo‐seq data. Fig. S2. Read densities of ribosome profiling data. Fig. S3. Expression data of ncRNAs found with ribosome profiling that are potentially coding, confirmed by qPCR in GSC34 and VU598. Fig. S4. Non‐coding RNA expression using single cell RNAseq data. Non‐coding RNAs identified by ribosome profiling were analyzed for their expression in the single‐cell clusters of normal cells as well as tumor cells. Fig. S5. Lethal effect of riboseq‐identified ncRNAs. Fig. S6. Subgroup DE analysis on transcriptome and translatome level. Fig. S7. Radiation sensitivity GSCs. [file MOL2-19-716-s002.zip › FigureSS4AB(1).jpg]
